# A Field-to-Parameter Pipeline for Analyzing and Simulating Root System Architecture of Woody Perennials: Application to Grapevine Rootstocks

**DOI:** 10.34133/plantphenomics.0280

**Published:** 2024-12-11

**Authors:** Lukas Fichtl, Daniel Leitner, Andrea Schnepf, Dominik Schmidt, Katrin Kahlen, Matthias Friedel

**Affiliations:** ^1^ Department of General and Organic Viticulture, Hochschule Geisenheim University, Geisenheim, Germany.; ^2^ Forschungszentrum Juelich GmbH, Agrosphere (IBG-3), Juelich, Germany.; ^3^ Department of Modeling and Systems Analysis, Hochschule Geisenheim University, Geisenheim, Germany.

## Abstract

Understanding root system architecture (RSA) is essential for improving crop resilience to climate change, yet assessing root systems of woody perennials under field conditions remains a challenge. This study introduces a pipeline that combines field excavation, in situ 3-dimensional digitization, and transformation of RSA data into an interoperable format to analyze and model the growth and water uptake of grapevine rootstock genotypes. Eight root systems of each of 3 grapevine rootstock genotypes (“101-14”, “SO4”, and “Richter 110”) were excavated and digitized 3 and 6 months after planting. We validated the precision of the digitization method, compared in situ and ex situ digitization, and assessed root loss during excavation. The digitized RSA data were converted to root system markup language (RSML) format and imported into the CPlantBox modeling framework, which we adapted to include a static initial root system and a probabilistic tropism function. We then parameterized it to simulate genotype-specific growth patterns of grapevine rootstocks and integrated root hydraulic properties to derive a standard uptake fraction (SUF) for each genotype. Results demonstrated that excavation and in situ digitization accurately reflected the spatial structure of root systems, despite some underestimation of fine root length. Our experiment revealed significant genotypic variations in RSA over time and provided new insights into genotype-specific water acquisition capabilities. Simulated RSA closely resembled the specific features of the field-grown and digitized root systems. This study provides a foundational methodology for future research aimed at utilizing RSA models to improve the sustainability and productivity of woody perennials under changing climatic conditions.

## Introduction

Root system architecture (RSA) constitutes a pivotal factor in plant adaptability and resilience to the challenges posed by climate change [1–3]. RSA encompasses the spatial arrangement and dynamic development of roots within the soil matrix, a key determinant of resource acquisition efficiency and plant performance under resource-limited conditions (e.g., during drought episodes) [4–9]. In woody perennials, optimized RSA can confer substantial long-term adaptive benefits under changing environmental conditions [10–12]. Especially in cropping systems utilizing grafted plants such as grapevine (*Vitis vinifera* L.), leveraging rootstocks with advantageous RSA presents a viable approach to enhance the competitive ability for soil resources while simultaneously preserving market-adapted varieties [13]. Notwithstanding, the precise spatial patterns of RSA in most woody perennials, the conceptualization of RSA ideotypes, and the impact of individual root architectural traits under defined growing conditions remain largely elusive. In this context, the application of computational root growth models holds the potential to provide an integrated comprehension of advantageous traits, thereby ensuring the strategic choice of genotypes tailored to specific agronomic scenarios [14]. For instance, RSA ideotypes to enhance water acquisition under specific drought stress profiles have been proposed based on in silico simulations (e.g., [1,15–17]). Employing computational models to delineate the spatial and temporal dynamics of water absorption and transport by plant roots can further enhance the selection of specific ideotypes for breeding and the implementation of model-informed modifications to farming practices (e.g., evaluation of intercropping systems) [17–19].

A primary obstacle in modeling RSA of woody perennials is the scarcity of detailed knowledge about the dynamic RSA development of these species under natural, unconstrained growth conditions, coupled with the inadequacies of extrapolating results from controlled pot or rhizotron studies to field environments. This is further complicated by the inconsistency of genotype-specific parameter estimates across different phenotyping platforms, highlighting the need for integrating realistic environmental and developmental factors in RSA analyses [20]. However, field-based phenotyping strategies capable of delivering high-resolution, functionally annotated root datasets are still lacking. Common methodologies often require a trade-off between data precision and proximity to natural growing conditions [21,22]. Therefore, the development of innovative phenotyping and data wrangling approaches is imperative, enabling the parameterization of root growth models that reflect the spatial complexity of woody perennial RSA under field conditions. Ideally, RSA data should be stored in an interoperable format to ensure its compatibility with a multitude of root system modeling frameworks.

**Fig. 1.**
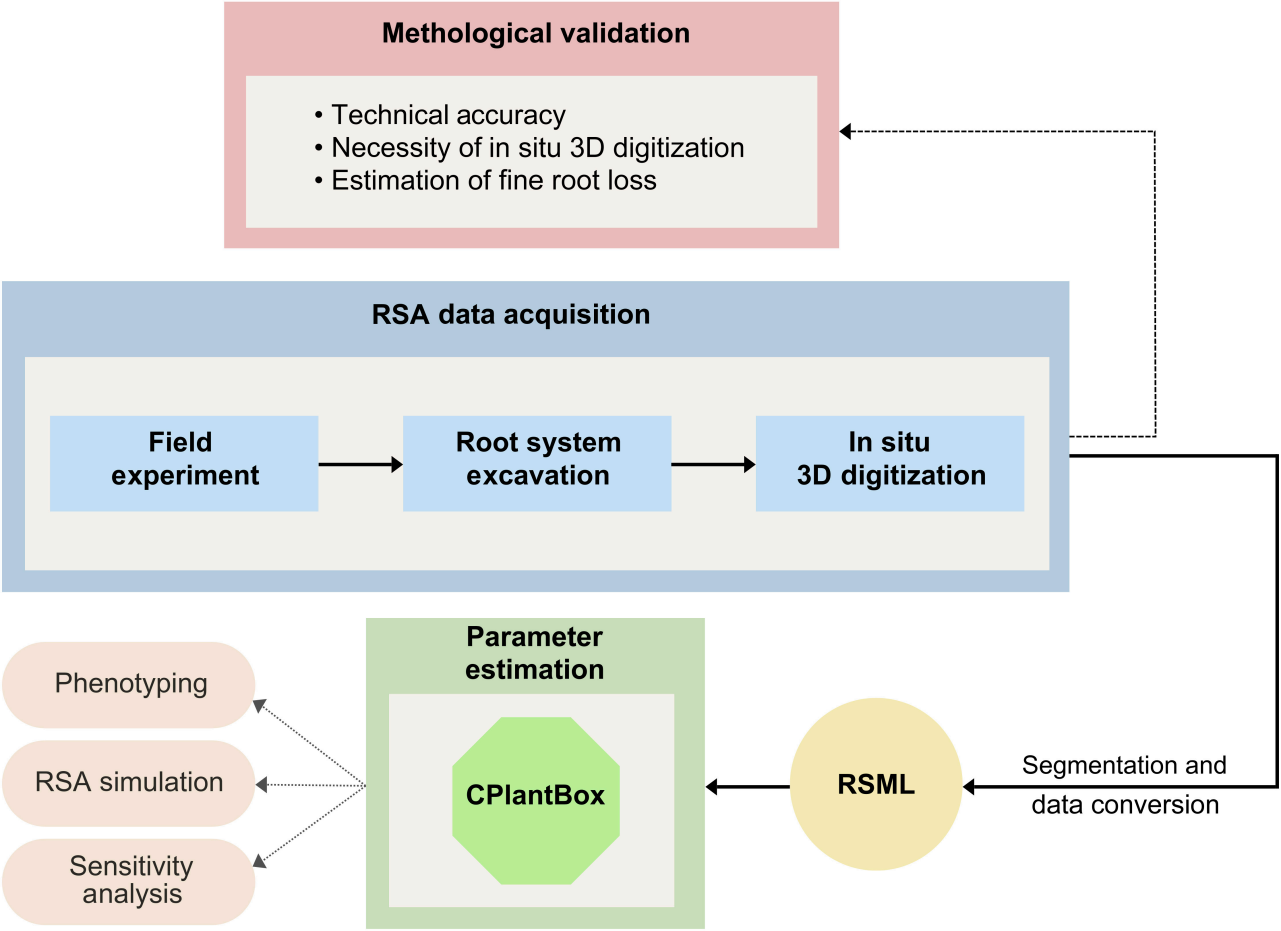
Schematic representation of the field-to-parameter pipeline outlining the process of RSA data acquisition through field experiments, root system excavation, and in situ 3D digitization, followed by the data transformation steps leading to parameter estimation in CPlantBox. The flowchart also delineates the methodological validation steps undertaken to test the technical accuracy, assess the necessity of in situ digitization, and estimate potential fine root loss during excavation. Arrows indicate the flow of data and analysis, highlighting the integration of RSA data with phenotyping and simulation outputs.

In this study, we present an integrative field-to-parameter pipeline for the analysis of RSA in woody perennials, with a particular focus on grapevine rootstock genotypes (Fig. 1). This approach combines field excavation and in situ 3-dimensional (3D) digitization, enabling comprehensive analysis of entire root systems while preserving the natural root–soil interface. A central element of our methodology is the transformation of RSA data into an interoperable format, specifically employing the root system markup language (RSML), which facilitates seamless integration into root modeling platforms [23]. In this context, we leverage CPlantBox, a whole-plant modeling framework, to interpret and simulate the complex dynamics of root growth [24]. This pipeline not only aids in detailed root system phenotyping and estimation of root growth model parameters but also paves the way for future ideotyping focused on enhancing water uptake efficiency, a key factor for plant resilience in the face of climate change.

## Materials and Methods

### Experimental design and plant material

Experiments were conducted in a field trial set up at the vineyards of the Department of General and Organic Viticulture, Hochschule Geisenheim University, Germany (latitude 49°59′16″N, longitude 7°56′56″E). A new vineyard was established in May 2023 according to good viticultural practice, using grafted grapevine cuttings. Cuttings derived from grafting the scion variety “Riesling” (*Vitis vinifera* L.; clone *N90*) onto the 3 distinct rootstock varieties “101-14 Millardet et de Grasset” (“101-14”) (*Vitis riparia* × *Vitis rupestris*; clone *3*), “Selection Oppenheim 4” (“SO4”) (*Vitis berlandieri* × *Vitis riparia*; clone *31 OP*), and “Richter 110” (“R110”) (*Vitis berlandieri* × *Vitis rupestris*; clone *152*). Grafted cuttings were 1 year old at planting, and adventitious roots were cut back to a length of approximately 10 to 15 cm before planting. Plantation was carried out according to common practice with an intervine distance of 1 m and a row width of 2 m, using a mechanical and GPS-supported (Global Positioning System) planting machine. The experiment was laid out as a fully randomized, complete block design with 4 blocks, 2 replicates per rootstock genotype and block, and 10 vines per replicate. The trellis system was created with metal-free materials only (wood posts, bamboo planting sticks, plastic wires) to avoid electromagnetic interference during 3D digitization (see the “3D digitization” section).

In addition to the main experimental setup, a parallel exploratory study was conducted by grafting the same scion clone “Riesling” onto the rootstock genotype “Cina” (*Vitis berlandieri* × *Vitis riparia* × *Vitis cinerea*; clone *N401*). Although these vines were planted in close spatial proximity to the primary experiment, the “Cina” rootstock was not integrated into the complete randomized block design. Instead, it was planted solely for the purpose of technical validation and to test the methodological procedures of our study (see the “Methodological accuracy” section).

### Measurements

Measurements were conducted in July and November 2023. At each time, 24 root systems (8 vines per genotype, one vine per replicate) were fully excavated and digitized in situ. For the experimental observations, only grapevines were selected, which were flanked by 2 vital neighboring vines, to ensure natural competitive dynamics among root systems. Root diameters (except fine roots) were measured using a caliper. Experiments for estimating the method’s accuracy were carried out in September 2023.

### Root system excavation

The excavation of the complete root systems of individual grapevines was undertaken by using manual techniques and small handheld devices, with an emphasis on the careful excavation of all root components, minimizing both destruction and loss of spatial structure. The vines, initially established as rooted cuttings, were planted so that the stem’s base was located approximately 20 cm below the soil surface, which also marked the depth of adventitious root initiation. Excavation commenced with the removal of the uppermost 10-cm layer of soil over an area of approximately 1 m^2^ using a spade (Fiskars, Espoo, Finland). A trench, approximately 1 m from the vine, was then dug to a depth of about 1.50 m using an excavator (Bobcat 19 mini excavator, Bobcat Company, West Fargo, USA) equipped with a 40-cm-wide bucket. This trench allowed for an ergonomic working position at chest height without cutting roots, facilitating a thorough and comfortable excavation process. To stabilize the vine and maintain its original position relative to the soil surface, a horizontal wooden framework was secured at the vine’s grafting point. Soil removal proceeded manually, from the stem base to the adventitious root zone, with the primary tool being a weeding trowel (Gardena, Ulm, Germany), which allows for gentle soil loosening around root structures. When necessary, a brush was used to carefully expose individual roots under dry conditions, avoiding any potential damage to fine root structures. Each adventitious root, along with lateral and fine roots, was excavated with precision, exposing roots in their natural spatial orientation within the soil matrix. Horizontally growing roots were fully exposed before excavating deeper roots to prevent structural disorientation. Throughout this process, all tools were chosen to avoid any adverse impact on the roots and surrounding soil structure. This careful handling allowed roots to retain their natural positions, ensuring accurate 3D digitization later on. Where accidental root severance occurred, roots were temporarily reattached with tape to facilitate continuity in the documentation and digitization process.

### 3D digitization

**Fig. 2.**
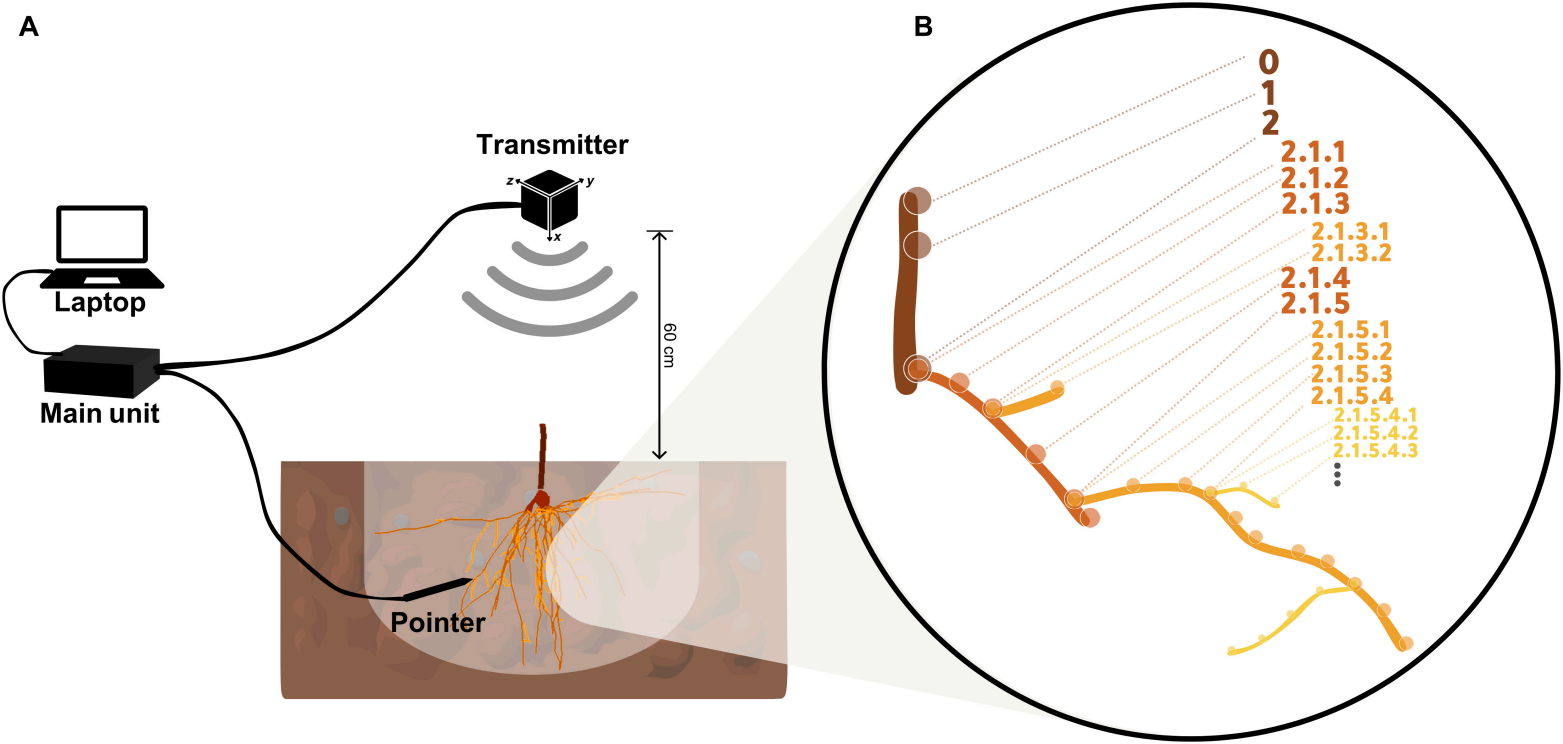
Technical setup and digitization protocol for in situ 3D digitization of root systems in original soil position after excavation. (A) Technical field setup with the main unit connected to a laptop, and an electromagnetic transmitter positioned 60 cm above the soil surface, detailing the configuration for recording root coordinates with a pointer. (B) Magnified view of the digitization process, showing the systematic assignment of unique IDs to digitized points, including double points at branching nodes to maintain the integrity of the root’s topological structure.

Root systems were digitized utilizing a Fastrak 3D digitizer (Polhemus, Colchester, USA). This device, operating on electromagnetic principles, comprises a main unit, a transmitter, and a pointer. The transmitter generates a low-frequency electromagnetic field in which the spatial coordinates of the pointer tip can be recorded. In the context of in situ digitization, a custom frame was positioned above the root system to hold the transmitter at an elevation of approximately 60 cm above the soil surface, ensuring that the emitted electromagnetic field was directed toward the soil, with the *x* axis pointing vertically downward (Fig. 2A). In this study, in situ digitization refers to the process of capturing the 3D coordinates of root systems while the roots remain in their natural position within the soil matrix. This approach is crucial for accurately reflecting the RSA as it exists in the field, trying to ensure that the spatial orientation of roots is maintained throughout the process.

Each root system was digitized following a standardized protocol (Fig. 2B). Digitization started with 3 points (0, 1, 2) on the stem, representing the grafting point, the height of the soil horizon, and the basal node of the cutting, respectively. Subsequent steps included systematic digitization of individual adventitious roots up to points of lateral branching, followed by the tracing of lateral roots through successive orders of branching. This procedure was repeated for each root, ensuring thorough coverage and detailed documentation of the topology of the root system.

In general, single roots were digitized with a number of points sufficient to delineate either a change in growth direction or the presence of branching points. Branching points were recorded twice to indicate that the first point of a lateral root is identical to the last point on the lower-order root (for example, in Fig. 2B, points 2.1.3 and 2.1.3.1). This ensures an accurate reconstruction of the root system topology. Basically, all roots visible after excavation were digitized, including fine roots with a length of minimum 0.5 cm. Functional annotation of the recorded coordinates was carried out simultaneously by using the DigiTool software (customizable research software, [25]), allowing the direct assignment of topological information to each point. For further details on the root system excavation and digitization process, including visual representations and step-by-step explanations, please refer to the guideline provided in the Supplementary Materials.

### Methodological accuracy

The digitization technique applied in this study, while previously utilized in other research contexts (for example, [26]), necessitated an evaluation of its accuracy. This need was particularly pronounced due to the novel field application of the technology and the potential interference from soil. Furthermore, an assessment of the necessity of conducting in situ digitization, as well as the destructiveness of the method, was considered essential to the study’s methodology.

#### Estimation of technical accuracy and human error

To evaluate the precision of the digitization technique in the context of in situ application, a standardized object with known dimensions was digitized using the same field setup as for root system digitization. Digitization was repeated in 30 randomly chosen locations and orientations within the electromagnetic field. In each case, 3 specific points were digitized to represent 2 segments, measuring 5 and 10 cm in length. Digitization of the object was conducted within an excavation pit after root system extraction, in order to closely replicate the natural positioning of root segments within the soil matrix. This included maximizing the variation in the *x*, *y*, and *z* coordinates relative to the transmitter and extended up to a maximum distance of 1.80 m from the transmitter. Additionally, to further ascertain the method’s accuracy and its susceptibility to operator-induced errors (e.g., hand or root movement during the digitization process), the deviation of double points within the digitization protocol (i.e., root branching points) was analyzed.

The methods’ accuracy was assessed by comparing the Euclidean distances computed between digitized points against the known distances of the object being digitized. Operator-induced error was quantified by calculating the Euclidean distances between double-digitized points and by measuring the deviation from an expected value of zero. The root mean square error (RMSE) was used as primary measure to summarize the overall precision of the method, by calculating the square root of the averaged squared differences between the digitized lengths and the actual lengths:

$$\text{RMSE}=\sqrt{\frac{1}{n}\sum_{i=1}^{n}\;{\left(x_{1}-y_{1}\right)}^{2}}$$ (1)

In addition, the bias was assessed by calculating the average difference between digitized and true lengths:

$$\text{Bias}=\frac{1}{n}\sum_{i=1}^{n}\;{\left(x_{1}\right)}^{2}-\frac{1}{n}\sum_{i=1}^{n}\;{\left(y_{1}\right)}^{2}$$ (2)

The accuracy of the method was calculated as 1 minus the ratio of the RMSE to the known lengths. Correlation analyses were conducted to explore whether the accuracy of the method varies systematically with the distance to the transmitter.

#### Assessing the necessity of in situ digitization

Digitizing root systems in the field presents additional challenges compared to the application under controlled laboratory conditions. These include technical and logistical difficulties (e.g., power supply in vineyards or electromagnetic interference caused by metal objects like trellis systems) and uncertainties or the need for flexibility with respect to weather conditions, given that the technique is not operable in rain. Therefore, it is crucial to assess whether in situ digitization is essential for accurate representation of natural RSA, or whether digitization can be effectively performed in a laboratory setting after excavation. A key determinant in this context is the extent to which the roots are sufficiently rigid to maintain their natural position and structure after excavation. To evaluate the significance of in situ digitization, 3 root systems of the “Cina” rootstock were excavated. These were digitized in the field following the described protocol, and then re-digitized under laboratory conditions in a hanging state. This approach enables the assessment of the extent to which removal of the root systems from the soil impacts the loss of architectural structure. For this purpose, architectural parameters of both digitization scenarios were compared, facilitating the determination of the necessity for in situ digitization of such large root systems of woody perennials.

#### Estimation of fine root loss due to excavation method

Despite extremely careful excavation by hand, and the aim to keep fine roots intact, some degree of loss of fine roots or fine root length is inevitable during the excavation of root systems. Quantifying the loss attributed to the excavation method is essential in order to estimate the need for supplementing excavated root systems with the simulation of fine root structure through computational root growth models, which may also incorporate data from nondestructive and high-resolution phenotyping methods with restricted root growth, such as rhizotrons. To estimate this degree of root loss, 2 more root systems of the “Cina” variety (located adjacent to the vines used for structure loss estimation) were flushed out the soil using water. This involved setting up a water tank and pump in the vineyard and gradually washing away the soil layers with water until the entire root system was exposed. This process was carried out using low water pressure to prevent damage to fine roots by the water jet. The exposed root systems were then digitized in a hanging state, facilitating a direct comparison with the excavated root systems to estimate the loss of fine roots or underestimation of fine root length resulting from the excavation process.

### Data processing

Digitization using the DigiTool permits the export of acquired 3D-RSA data as .txt files with a straightforward structure. In these files, the *x*, *y*, and *z* coordinates are associated with an organ type and a unique ID, which delineates the hierarchy of the points and thus describes the topological structure of the data. The .txt files of the digitized root systems were imported into R using a customized script, and the points were interconnected according to their topology to create line segments. Root lengths were calculated as Euclidean distances between pairs of points, followed by the transformation of the 3D data into the RSML format. This format organizes RSA data in a standardized XML structure, which is supported as an interoperable format by various phenotyping and modeling platforms [23]. The RSML format arranges root segments in a nested manner (i.e., topological assignment) and includes information on the geometry (i.e., 3D coordinates) of the root segments, along with additional properties assigned to each segment (e.g., root diameter). The RSML files of the root systems were subsequently analyzed using the modeling framework CPlantBox, which is a whole-plant modeling framework that enables the evaluation and simulation of plant architectures [27]. Basic evaluations can be swiftly and effectively conducted using the CPlantBox *viewer* and *estimator*, both of which are graphical user interfaces (GUIs). The CPlantBox *viewer* allows users to visualize root system structures and related properties in 3D, while the *estimator* facilitates parameter extraction and analysis.

### Model adaptation and parameterization

**Fig. 3.**
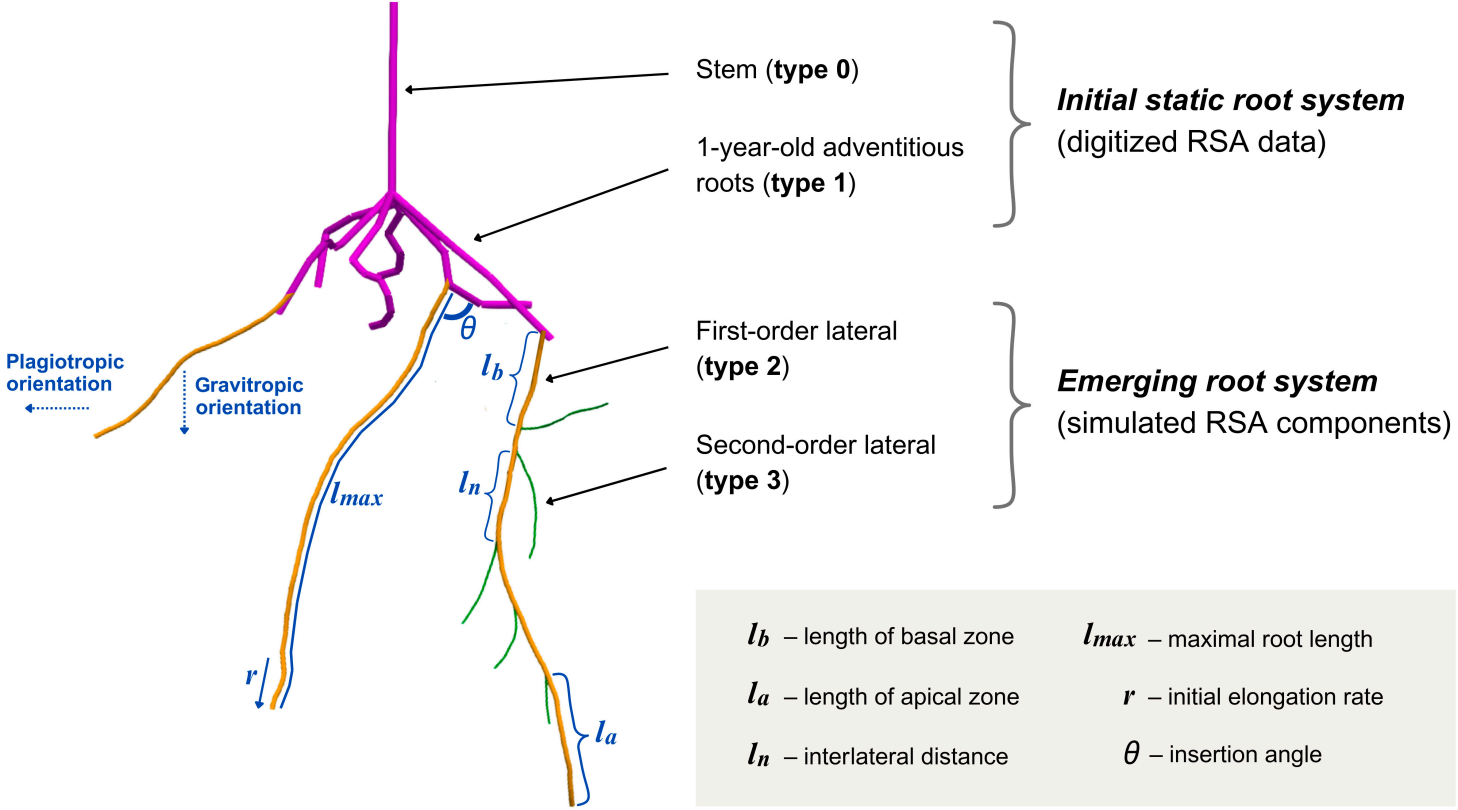
Schematic representation of our approach for grapevine RSA simulation. The initial static root system, derived from digitized RSA data, includes the stem (type 0) and 1-year-old adventitious roots (type 1). The emerging root system, composed of simulated RSA components, includes first-order laterals (type 2) and second-order laterals (type 3).

In this study, we adapted the FSPM CPlantBox to simulate the RSA of grapevine rootstocks by extending the original model code and parameterizing it based on our 3D RSA measurements. The adaptations involved 2 primary modifications. First, unlike the original CPlantBox version, which initiated growth from a seed, we extended the code to allow growth from an initial static root system—specifically, a 1-year-old woody adventitious root system. This adaptation enabled us to model the root growth of grapevine realistically, starting from a rooted cutting rather than a germinating seed. Second, we modified the tropism function to better replicate the directional root growth patterns observed in our field studies, capturing the genotype-specific tropic responses characteristic of different rootstock genotypes. Regarding parameterization, CPlantBox did not have an existing dataset for grapevine root architecture, necessitating the creation of new parameters for each genotype. All parameters, including maximal root length, initial elongation rates, and branching characteristics, were derived from our 3D RSA measurements to accurately reflect the growth behavior of different rootstock genotypes. Growth parameters were calculated using the CPlantBox *estimator*. Structural analysis of our 3D RSA data led to the classification of roots into 3 types: type 1, representing the initially planted, woody, 1-year-old adventitious roots; type 2, the first-order roots that emerge after plantation from type 1 roots; and type 3, second-order roots or fine roots. This hierarchical structure allowed us to model the growth dynamics of grapevine rootstocks more precisely, with type 1 roots serving as a static foundation for subsequent root growth, as illustrated in Fig. 3. All model adaptations and parameterizations are described in detail in the following paragraphs.

#### Static root system initialization

Our model introduces an initial static root system to represent the 1-year-old adventitious roots of grapevines. These adventitious roots, pruned before planting, form the initial structural foundation for subsequent growth, as observed in our 3D RSA data. The static root system is characterized by its stability—it ceases to grow further after planting and is a static structure on which new lateral roots can be formed. To initialize the static system, we import an RSML file of a specific rootstock genotype containing the measured root structure data for type 1 roots. These measurements reflect the spatial configurations as observed in the field, incorporating the randomized positioning resulting from the mechanical planting process. The pseudocode below illustrates the core steps involved in initializing the static adventitious root system.

**def** initialize_static_root_system(rsml_file):

 data = load_rsml(rsml_file) *# load RSML root data of a specific rootstock genotype*

 static_initial_root_system = [ ] *# initialize an empty list tohold the static roots*

**for** root **in** data:

 **if** root.type == 1: *# only select adventitious roots (type 1)*

  static_root = create_static_root(nodes, root_lifetime=1.e6) *# extract the root   geometry* *and set root lifetime to a very large value (persistent roots)*

  static_initial_root_system.append(static_root)

**return** static_initial_root_system

In this initialization process, type 1 roots are imported directly from RSML data, which include root geometry and branching points. These roots are defined as having an effectively infinite lifetime, ensuring their persistence throughout the simulation and serving as the structural base from which type 2 lateral roots emerge according to predefined criteria.

#### Dynamic root growth parameterization

The dynamic growth of type 2 and type 3 roots is built upon the static adventitious root system, following general CPlantBox principles with further specific adaptations to suit our grapevine model. This involves the growth and emergence of type 2 lateral roots and their subsequent branching into type 3 roots, guided by a set of well-defined growth parameters. The pseudocode below describes the process of adding lateral roots to the static adventitious roots during the simulation. Each lateral root is defined by its type, emergence timing, and an emergence probability.

**def** add_laterals_to_static_root_system(static_initial_root_system):

 **for** root **in** static_initial_root_system: *# iterate through each root in the static root  system*

  **for** node_index **in** root.get_lateral_emergence_points(): *# set lateral with emergence   probability and timing*

   root.add_lateral(node_index, lateral_type=2, emergence_time=0., emergence_prob=1.0)

In our simulations, we chose that all type 2 lateral roots emerge simultaneously (emergence_time = 0.), as this approach is consistent with our observations; we did not identify an increase in the number of type 2 roots between 3 and 6 months after planting, indicating that these roots emerge predominantly in an initial phase after planting. The probability of emergence is set to 1.0, meaning that all lateral emergence points develop into full lateral roots, because our goal is to reconstruct the original RSA as observed in our field data, letting lateral roots emerge from all points where roots were originally present within the static initial root system. Key growth parameters for type 2 and type 3 roots—including maximal root length ($l_{\max}$), initial elongation rate (*r*), basal and apical zone lengths ($l_{b}$ and $l_{a}$), interlateral distance ($l_{n}$), insertion angle (θ), and root radius (*a*)—were derived from our 3D-digitized RSA data and parameterized for each genotype and root type. Table 4 provides an overview of the model input parameters for each rootstock genotype. In CPlantBox, each parameter is defined by a mean and an SD, enabling stochastic variability among individual branches and thus better approximating biological diversity. Maximal root length ($l_{\max}$) governs the potential final length of each root, while the initial elongation rate (*r*) determines the speed at which roots extend after emergence. Basal and apical zone lengths ($l_{b}$ and $l_{a}$) define regions at the root base and tip where no lateral roots are formed. The interlateral distance ($l_{n}$) controls the spacing between lateral root emergence points, a crucial factor in simulating branching density. The insertion angle (θ), calculated from the angle formed between the mother root and lateral root, influences the directional growth and spatial configuration of the branches. Finally, root radius (*a*) is used to determine the hydraulic properties of the root system and plays an important role in shaping the overall architecture. To introduce biological variability, the dynamic parameters are sampled from a truncated normal distribution during each simulation, avoiding unrealistic negative values while reflecting natural variations observed among genotypes. This approach ensures that each simulated root system represents a possible realization of growth for the given parameter set. Together, the static root system and the dynamic growth processes—including the emergence and elongation of type 2 and type 3 roots—allow our model to simulate realistic grapevine root development, beginning from a mechanically planted cutting and expanding through dynamic branching driven by genotype-specific traits.

#### Probabilistic tropism implementation

From one root segment to the next, CPlantBox computes the new growth direction of a growing root tip by adding an axial angular change to the previous growth direction. This angular change is drawn randomly from *N*(0, σ), where σ is the SD of random angular change. When there is a preferred growth direction (tropism), a new random growth direction is drawn *N* times, and the new growth direction is taken to be the one closest to the preferred one. The parameter *N* is the tropism strength. The original CPlantBox allows for one preferred growth direction, while our field observations showed mixed occurrence of gravitropic and plagiotropic growth patterns observed in type 2 roots. Some type 2 roots exhibited gravitropism, growing downward toward deeper soil layers; others followed plagiotropic patterns, growing horizontally within the topsoil. To account for this variation, we allowed for variability in growth behavior within type 2 roots. The probabilities for gravitropic or plagiotropic growth were determined for each genotype based on the spatial distribution of type 2 root tips (see Fig. S7). For instance, 15% of root tips of long type 2 roots (root length > 40 cm) were present in the top soil (soil depth < 50 cm) for the genotype “101-14”, resulting in a 15% probability of plagiotropic behavior. In the new adapted CPlantBox model, we choose a new growth direction for type 2 roots by generating a random value from a uniform distribution over [0,1] after each root segment length (*dx*). If the value is below the threshold defined for gravitropism (e.g., 0.85 for genotype “101-14”), the root is assigned gravitropic growth. Otherwise, plagiotropic growth is applied. This probabilistic component was integrated into the original CPlantBox tropism function, as illustrated in the pseudocode below.

**def** tropismObjective(pos, old, alpha, beta, dx, root):

 new_direction = (old * rotX(beta)) * rotZ(alpha)

 **if** subType == 2: *# application solely to root type 2*

  random_value = np.random.rand()

  **if** random_value < 0.85: *# genotype-specific likelihood*

   **return** new_direction.column(0).z *# selection of largest negative z-coordinate*

  **else**:

   **return** abs(new_direction.column(0).z) *# selection of z-coordinate closest to zero*

Details on the underlying tropism mechanism can be found in the CPlantBox documentation [24,27,28]. In brief, this function evaluates the current root tip position (*pos*) and orientation (*old*), applies axial (*alpha*) and radial (*beta*) angular changes using rotation matrices, and computes the new growth direction by a random optimization algorithm for different angles. For roots exhibiting gravitropism, the objective function minimizes the *z*-coordinate of the new direction, ensuring downward growth. In the case of plagiotropic roots, the absolute *z*-coordinate is minimized, keeping the growth trajectory close to the horizontal plane. This random assignment of tropism allows the simulation to reflect the natural variability in root growth patterns observed in our data, providing a more realistic representation of genotype-specific RSA development.

### Root hydraulic property estimation

#### Root cross-sectioning and estimation of root hydraulic conductivity

Following the excavation of 24 grapevine individuals 6 months after planting, root samples were systematically collected for anatomical analysis. Type 1 roots, defined as 1-year-old adventitious roots, were sampled at a standardized distance of 5 cm from the trunk base. Additionally, type 2 roots, classified as primary lateral roots, were sampled at the suberized base zone approximately 20 cm from the emergence point on the main root, as well as at the unsuberized distal zone. The harvested root samples were washed under a stream of water, fixed in an AFA solution (alcohol, formalin, and glacial acetic acid) for 12 hours, and subsequently preserved in 80% ethanol at room temperature, stored within Eppendorf tubes. Cross-sectioning was performed by an external laboratory (Morphisto GmbH, Offenbach am Main, Germany). In brief, 5-μm root cross-sections were prepared with a sharp blade microtome, then stained with toluidine blue to enhance contrast for microscopy, and mounted onto glass slides.

The prepared sections were imaged using a digital camera affixed to a light microscope. For the purpose of this study, 33 cross-sections with high diameter variability, corresponding to 11 per genotype (comprising 4 samples from main roots, 4 from suberized lateral roots, and 3 from unsuberized root tips), were digitally analyzed with image processing software (Digimizer 6.3.0, [29]). Each cross-section was assessed for xylem vessel count, with the lengths of the minor and major elliptical axes of each xylem vessel measured.

Theoretical axial hydraulic conductivity (${\mathrm{Kh}}_{\text{theo}}$) was calculated employing the Hagen–Poiseuille equation, adapted for elliptical xylem vessels [30]. This equation is represented as:

$${\mathrm{Kh}}_{\text{theo}}=\frac{\pi }{64\eta }\sum_{i=1}^{n}\;\left(\frac{a_{i}^{3}b_{i}^{3}}{a_{i}^{2}+b_{i}^{2}}\right)$$ (3)

where ${\mathrm{Kh}}_{\text{theo}}$ $\left(m^{4}s^{-1}\mathrm{MP}a^{-1}\right)$ is the theoretical axial hydraulic conductivity of conduits in a cross-section, η is the dynamic viscosity of water at 20 °C ($1.002\times 10^{-9}\text{MPas}$), and n is the number of xylem conduits in a cross-section; $a_{i}$ and $b_{i}$ correspond to the minor and major diameters of the elliptical xylem vessel.

#### Hydraulic model implementation

The resulting theoretical hydraulic conductivity (${\mathrm{Kh}}_{\text{theo}}$) data were subject to nonlinear least squares modeling to establish correlations with individual root diameters [31]. Analysis was executed separately for each rootstock genotype, yielding genotype-specific functional relationships, facilitating direct integration into our root growth model for diameter-dependent calculation of the theoretical axial hydraulic conductivity for individual root segments. Values regarding radial hydraulic conductance (${\mathrm{Lp}}_{r}$; $m\;s^{-1}\;{\mathrm{MPa}}^{-1}$) of contrasting root sections—including fine roots, suberized root segments, and woody root portions—were derived from existing literature. In particular, values published by Gambetta et al*.* [32,33] and Cuneo et al*.* [34] provided a comprehensive reference, allowing for the adaptation of ${\mathrm{Lp}}_{r}$ values pertinent to the range of grapevine root types present within our study, although no rootstock genotype-specific values were available. These adaptations were pivotal in constructing a more complete and accurate representation of root water transport dynamics within our model.

Based on root hydraulic properties, we computed the standard uptake fraction (SUF) for each root segment, determining the relative contribution of RSA compartments to root water uptake under uniform soil water potential conditions, and the root system conductance $\left(K_{\mathrm{rs}}\right)$ [35,36]. The SUF was calculated taking into account genotype, root type, and root diameter. Conductivities were chosen based on our empirical observations as well as published data and included parameters for root radial and axial conductance with root age-dependent adjustments, ensuring accurate representation of root water uptake capabilities over time.

### Data and statistical analysis

Data analysis, including the import of digitized 3D RSA data, root system reconstruction, segmentation, and transformation into RSML format, was conducted using R (version 4.4.0, [37]) with the GUI RStudio (version 2024.04, [38]). Global RSA parameter estimation was performed using the ArchiDART package in R (version 3.4; [39]). Root system visualization was accomplished with ParaView (version 5.12.0, [40]). Statistical analyses were also conducted using R (lme4 and lmerTest packages, [41,42]), employing a linear mixed effects model to evaluate the effects of rootstock genotype and time on various RSA parameters. Our model incorporated rootstock genotype, time, and their interaction as fixed effects while accounting for random effects associated with block. Post hoc pairwise comparisons between rootstock genotypes for parameters significantly affected were conducted using the least significant difference test (lsmeans package, [43]).

## Results

### Methodological accuracy

#### Estimation of technical accuracy and human error

**Fig. 4.**
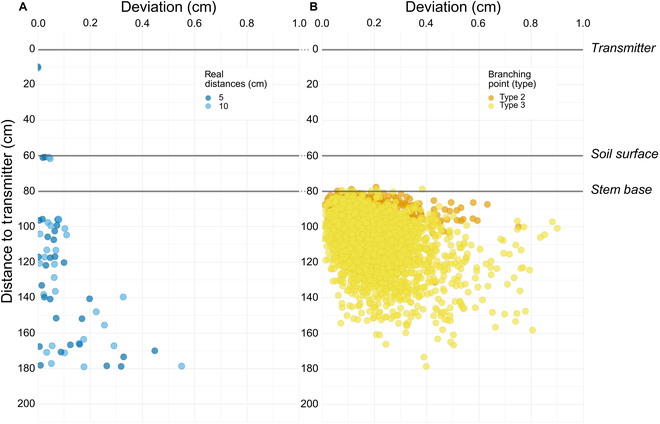
Validation of the digitization accuracy and assessment of human error. (A) Deviation from predefined distances (5 cm and 10 cm lengths) of a known object in relation to its distance from the transmitter, quantifying the technical accuracy of the digitization method. Each point (*n* = 60) represents an individual measurement, with colors indicating the lengths of digitized segments. (B) Deviation of double-digitized points (*n* = 4,320), which reflect root system branching points and human error during the digitization process, with colors indicating different root branching types. The gradient from soil surface to stem base on the *y* axis and the density of points indicate the error distribution across the digitization field.

The assessment of the method’s technical accuracy (Fig. 4A) revealed an overall bias of 0.075 cm, suggesting a negligible overestimation. Notably, the bias increased with the distance to the transmitter, from 0.039 cm for distances under 100 cm to 0.144 cm for distances beyond 150 cm. The overall RMSE was 0.154 cm, with accuracies of 98% for 10-cm segments and 97% for 5-cm segments, indicating a high level of precision. The RMSE values rose with increasing distance to the transmitter, reaching up to 0.231 cm for a distance greater than 150 cm.

In assessing human error through the deviation of double-digitized points (Fig. 4B), we observed a mean deviation of 0.182 cm. This deviation increased with distance to the transmitter (from 0.155 cm at less than 100 cm to 0.351 cm at distances greater than 150 cm), suggesting that operator-induced errors have a more pronounced effect than the method’s inherent technical accuracy. However, the deviation, even at the farthest measured distances, remained below half a centimeter, underscoring the method’s overall reliability. Linear regression revealed that only approximately 12.43% of the variance in digitization discrepancy can be attributed to the variation in distance from the transmitter ($R^{2}$ = 0.1243), indicating a systematic, albeit small, deviation in the digitization of root branching points.

#### Assessing the necessity of in situ digitization

**Fig. 5.**
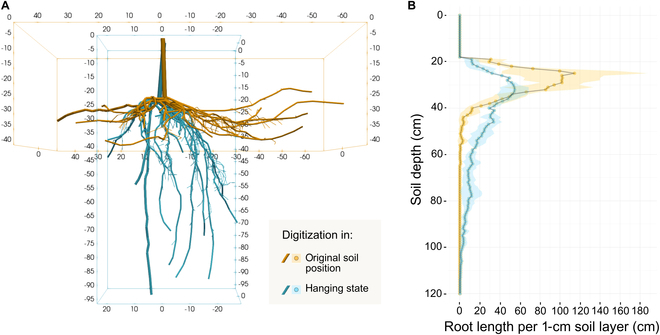
Comparative visualization of root system digitization methods and their impact on root architecture representation. (A) 3D reconstruction of the same “Cina” root system digitized using 2 different methods, highlighting the structure loss when digitized after removal from the soil (ex situ, in a hanging state) versus the natural positioning within the soil matrix (in situ). The original soil position is indicated in orange, while the hanging state is shown in blue. (B) Root length profile comparison, where the main trend lines represent the average root length per 1-cm soil layer, and the shaded areas denote the SD among measurements from 3 “Cina” root systems, each digitized twice.

In our comparative analysis of root system digitization techniques, we observed pronounced differences in the RSA representation when comparing in situ digitization within the soil matrix to that of a hanging state under controlled laboratory conditions. Figure 5A showcases the architectural differences of an exemplary root system digitized by both methods. The root length profiles of all 3 root systems digitized and re-digitized for this experiment are presented in Fig. 5B.

A distinct differentiation in RSA between the 2 methods is evident, as the graphical representation indicates a consistent pattern, further underlined by the fact that identical root systems were digitized: In situ digitization captures the RSA with greater fidelity to its natural orientation and distribution within the soil matrix. Notably, the in situ digitization of the “Cina” rootstock captured a shallower average rooting depth (50 cm versus 80 cm) and a more accurate representation of lateral spread (138 cm versus 46 cm). Root systems digitized after removal from the soil (ex situ) exhibited clear signs of structural deformation, resulting in narrower root systems and an overestimation of root volume in deeper soil layers (>40cm). Furthermore, ex situ digitization consistently led to a reduction in the 3D root system convex hull volume by approximately factor 2.5, with reductions in the horizontal spatial dimensions of 84.3% in the *XY* plane and 10.8% in the *XZ* plane. This illustrates the extent of root system volume loss and deformation when roots are digitized in a hanging state. Additionally, the mean length of first-order laterals (type 2) and fine roots (type 3) decreased by 15.8% and 5.9%, respectively, underscoring the susceptibility of finer, nonwoody root structures to loss, damage, or shrinkage during the removal process. These findings underscore the critical importance of in situ 3D digitization for preserving the natural size, orientation, and distribution of root systems, ensuring a more accurate representation of RSA under field conditions.

#### Estimation of fine root loss due to excavation method

Our findings demonstrate distinct differences between the excavation and washout methods for estimating fine root loss (Table 1). The excavation method, while conducted with utmost care to preserve root integrity, resulted in lower total fine root lengths and shorter single fine roots. The washout method, utilizing a gentle water jet, revealed more extensive fine root structures, highlighting the methodological limitations of excavation in preserving complete fine root architecture. However, our findings emphasize the excavation method’s relative accuracy in estimating the number of fine roots compared to the washout method. This accuracy in count, despite the loss in length, is critical for obtaining reliable interlateral distances, which serves as valuable model input parameters.

**Table 1.** Total and average lengths of fine roots as well as the count of fine roots per root system obtained by excavation and washout extraction methods (2 plants per method). The results underscore a notable disparity, with the washout method revealing a more extensive fine root network than excavation, reflecting the latter’s inherent limitations in preserving the entirety of fine root structure during manual excavation.

Plant	Method	Total fine root length (cm)	Single fine root length (cm)	Total number of fine roots
1	Excavation	640.7	5.3 ± 4.2	121
2	Excavation	689.8	4.6 ± 3.8	149
3	Washout	1,776.9	12.3 ± 10.6	145
4	Washout	1,449.7	10.5 ± 8.0	138

### Quantitative analysis of RSA

Our analysis of RSA across the 3 grapevine rootstock genotypes (“101-14”, “SO4”, and “R110”) effectively demonstrates the efficacy of our methodology in capturing genotypic differences and the dynamic nature of RSA development over time (Table 2). We observed significant changes in several RSA parameters from T1 (3 months after planting) to T2 (6 months after planting), with all genotypes showing substantial growth within the first growing season (e.g., increasing total root system length, *P* < 0.001). The increase in total lateral root length from T1 to T2 across all genotypes (*P* < 0.001), without significant differences between them (*P* > 0.1), suggests a uniform growth pattern in terms of lateral root development over time. Significant temporal effects were also evident as genotypic differences became more pronounced at T2, underscoring the importance of developmental stage in RSA assessment. The results highlight that time is a critical factor influencing RSA characteristics. The significant interaction effects between rootstock genotype and time for several RSA parameters (e.g., root system height) indicate that these genotypes not only differ intrinsically but also respond differently to temporal growth dynamics. The relative stability of the mean length of type 1 roots (1-year-old adventitious roots) over time supports the assumption that these roots do not continue to elongate after planting. This finding, consistent across all genotypes, aligns with the understanding that once planted, the primary structure of these roots remains largely unchanged.

**Table 2.** Summary of RSA parameters of the 3 studied grapevine rootstock genotypes (“101-14”, “SO4”, and “R110”) measured at 2 time points (T1: 3 months after planting and T2: 6 months after planting). Values are presented as mean ± SD. Parameter estimation was performed based on digitized 3D data from a total of 48 root systems (8 per genotype and time point). Statistical significance was assessed using a mixed effects model with genotype and time as fixed factors and block as a random factor. Significance levels are indicated as follows: n.s. (not significant), ^.^ (*P* < 0.1), * (*P* < 0.05), ** (*P* < 0.01), *** (*P* < 0.001). Letters (a, b, c) denote significant differences between genotypes based on post hoc pairwise comparisons. No significant effects associated with block were detected for any of the parameters.

	101-14			SO4			R110					
Parameter	T1	T2		T1	T2		T1	T2		Rootstock	Time	Interaction Rootstock:Time
Total root system length (cm)	1,106 ± 160	1,986 ± 352	–	1,025 ± 335	2,032 ± 452	–	1,092 ± 317	1,982 ± 474	–	n.s.	***	n.s.
Total number of lateral roots (–)	108 ± 26	148 ± 27	b	85 ± 36	126 ± 31	a	85 ± 38	93 ± 17	a	***	***	n.s.
Total lateral root length (cm)	1,083 ± 160	1,962 ± 352	–	1,002 ± 334	2,011 ± 452	–	1,070 ± 316	1,959 ± 474	–	n.s.	***	n.s.
Total number of type 1 roots (–)	11 ± 3	7 ± 1	–	10 ± 4	9 ± 3	–	9 ± 4	6 ± 2	–	n.s.	***	n.s.
Total number of type 2 roots (–)	37 ± 11	23 ± 9	–	33 ± 15	24 ± 6	–	45 ± 19	25 ± 6	–	n.s.	***	n.s.
Total number of type 3 roots (–)	61 ± 19	119 ± 23	c	43 ± 19	94 ± 26	b	31 ± 20	62 ± 13	a	***	***	n.s.
Total root length of type 1 roots (cm)	155 ± 70	80 ± 29	–	127 ± 48	102 ± 43	–	129 ± 45	67 ± 25	–	n.s.	***	n.s.
Total root length of type 2 roots (cm)	550 ± 131	861 ± 258	a	453 ± 142	903 ± 256	a	720 ± 217	1,210 ± 329	b	***	***	n.s.
Total root length of type 3 roots (cm)	267 ± 61	947 ± 189	b	215 ± 91	834 ± 216	b	149 ± 87	573 ± 175	a	***	***	*
Mean length of type 1 roots (cm)	14.4 ± 3.4	12.7 ± 6.2	–	13.3 ± 1.7	12.1 ± 2.5	–	15.0 ± 3.7	10.6 ± 1.5	–	n.s.	*	n.s.
Mean length of type 2 roots (cm)	15.6 ± 5.2	40.7 ± 10.1	ab	14.6 ± 3.2	40.3 ± 12.1	a	16.8 ± 3.4	48.3 ± 9.1	b	.	***	n.s.
Mean length of type 3 roots (cm)	4.5 ± 0.7	8.0 ± 0.9	–	5.1 ± 1.4	8.9 ± 1.0	–	5.3 ± 2.2	9.3 ± 2.5	–	n.s.	***	n.s.
Mean diameter of type 1 roots (cm)	0.27 ± 0.05	0.43 ± 0.08	b	0.25 ± 0.04	0.36 ± 0.06	a	0.035 ± 0.06	0.47 ± 0.06	c	***	***	n.s.
Mean diameter of type 2 roots (cm)	0.13 ± 0.01	0.23 ± 0.04	–	0.13 ± 0.02	0.20 ± 0.04	–	0.12 ± 0.02	0.22 ± 0.03	–	n.s.	***	n.s.
Mean lateral root diameter (cm)	0.12 ± 0.01	0.14 ± 0.02	a	0.12 ± 0.01	0.14 ± 0.01	a	0.15 ± 0.02	0.17 ± 0.02	b	***	***	n.s.
Surface area of type 1 roots (cm^2^)	108 ± 31	99 ± 19	–	84 ± 20	102 ± 34	–	116 ± 44	91 ± 29	–	n.s.	n.s.	n.s.
Surface area of type 2 roots (cm^2^)	223 ± 48	606 ± 167	a	175 ± 48	575 ± 191	a	282 ± 97	884 ± 265	b	***	***	*
Surface area of type 3 roots (cm^2^)	42 ± 10	149 ± 30	b	34 ± 14	131 ± 34	b	23 ± 14	90 ± 27	a	***	***	**
Total root surface area (cm^2^)	598 ± 104	1,094 ± 206	a	586 ± 135	1,125 ± 356	ab	629 ± 179	1,357 ± 346	b	·	***	n.s.
Total volume of type 1 roots (cm^3^)	8.4 ± 2.0	11.5 ± 2.9	ab	6.1 ± 1.3	9.8 ± 3.7	a	11.4 ± 4.3	11.5 ± 3.5	b	*	*	n.s.
Total volume of type 2 roots (cm^3^)	8.1 ± 1.8	38.6 ± 14.4	a	6.2 ± 2.1	34.0 ± 13.4	a	10.4 ± 4.9	58.4 ± 20.7	b	**	***	*
Total volume of type 3 roots (cm^3^)	0.5 ± 0.1	1.9 ± 0.4	b	0.4 ± 0.2	1.6 ± 0.4	b	0.3 ± 0.2	1.1 ± 0.3	a	***	***	*
Total root system volume (cm^3^)	94.4 ± 11.3	135.1 ± 23.9	a	95.1 ± 13.7	130.6 ± 22.3	a	99.8 ± 15.4	158.8 ± 30.9	b	*	***	n.s.
Root system height (cm)	57.9 ± 4.9	92.6 ± 12.5	a	54.8 ± 4.9	92.2 ± 15.2	a	58.5 ± 4.8	116.7 ± 14.9	b	***	***	***
Root system width (cm)	58.5 ± 11.6	96.8 ± 17.7	b	64.3 ± 18.5	100.4 ± 27.9	b	51.0 ± 12.6	53.7 ± 15.3	a	***	***	**
Volume of the 3D convex hull (m^3^)	0.036 ± 0.018	0.140 ± 0.040	b	0.029 ± 0.014	0.185 ± 0.098	b	0.024 ± 0.008	0.080 ± 0.036	a	**	***	**

**Fig. 6.**
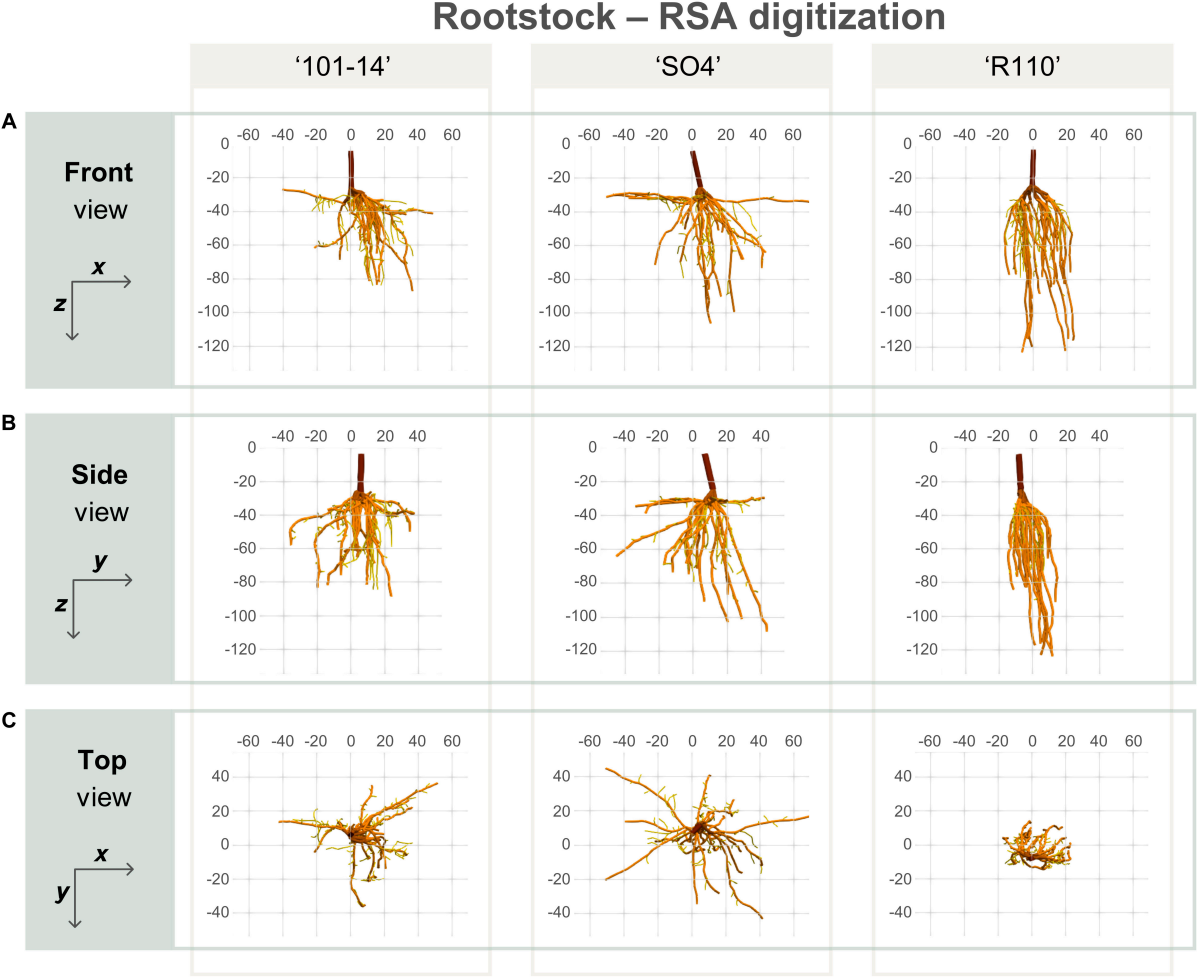
3D visualizations of representative root systems for each grapevine rootstock genotype studied, depicting the inherent differences in root architecture. (A to C) Front, side, and top views, respectively, illustrating the spatial orientation and distribution of roots in relation to the vineyard planting arrangement. The genotypes “101-14”, “SO4”, and “R110” are represented from left to right, with “101-14” being drought susceptible, “R110” being drought tolerant, and “SO4” exhibiting intermediate drought tolerance. The visualization axes are aligned with the vineyard layout: *x* axis parallel to the plantation row, *y* axis toward the inter-row space, and *z* axis indicating soil depth. Color coding within each root system denotes the stem and main roots in dark brown, lateral roots in light brown, and fine roots in yellow, showcasing the genotypic variability in root system structure relevant to water uptake and drought response.

Our methodology extends beyond classical RSA parameters like root lengths by utilizing 3D data to provide additional insights, such as measurements of root system height, width, and the volume of the convex hull. For instance, “R110” showed a unique growth pattern, characterized by the largest root system depth and the smallest 3D convex hull volume at T2, indicating a more vertically oriented growth strategy compared to the other genotypes, which exhibited more extensive lateral spread (Fig. 6). This enriched dataset enhances our understanding of the spatial complexity of root systems, and our methodology proves robust in detecting genotypic differences.

### Root length distribution and SUF

**Fig. 7.**
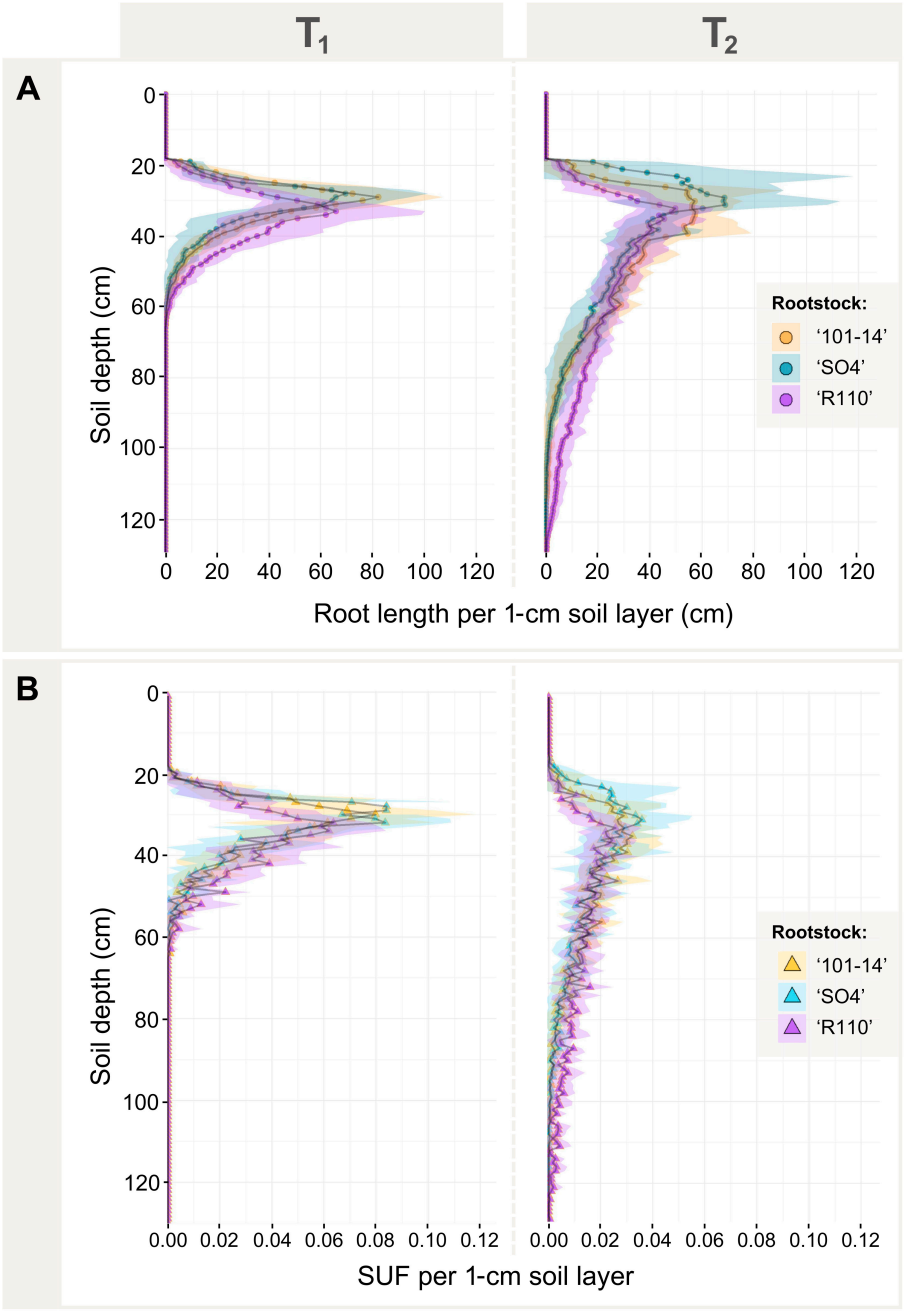
Root length distribution and simulated SUF by soil depth for the 3 grapevine rootstock genotypes (“101-14”, “SO4”, and “R110”) at 2 time points (T1: 3 months after planting, T2: 6 months after planting). (A) Root length per 1-cm soil layer (cm) based on digitized 3D data, showing the vertical distribution of root length at T1 and T2. (B) SUF per 1-cm soil layer, derived from model output based on our hydraulic RSA model, illustrating the distribution of water uptake potential with soil depth at T1 and T2. Points/triangles represent mean values per 1-cm soil layer, and shaded areas denote the SD among measurements.

The root length distribution and SUF of the 3 grapevine rootstock genotypes were analyzed with respect to soil depth, revealing significant genotypic and temporal differences, as depicted in Fig. 7. Between T1 and T2, root length per 1-cm soil layer varied considerably, with all genotypes showing substantial root growth extending into deeper soil layers. Initially, at T1, root systems were predominantly concentrated within the top 60 cm of soil. However, by T2, notable extensions into deeper layers were observed, particularly for “R110”, which exhibited the most significant increase in root length within the 61- to 90-cm and 91- to 120-cm soil layers (Fig. 7A).

Our SUF analysis, based on the assumption that soil is in hydrostatic equilibrium, further highlighted these genotypic differences in terms of potential water uptake distribution (Fig. 7B). At T1, the highest SUF values were observed in the top 60-cm soil horizons across all genotypes. However, by T2, a significant decrease in SUF was noted in the upper 30-cm horizon for all genotypes (*P* < 0.001), although no significant time effect was observed regarding root length in this soil compartment, representing our root age-dependent water uptake model. Particularly, “R110” indicated a shift in water uptake to deeper soil layers with root system age, as SUF values at T2 were significantly higher for “R110” in the deeper horizons (61 to 90 cm and 91 to 120 cm) compared to the other genotypes (*P* < 0.001). In the 31- to 60-cm soil horizon, SUF values remained relatively stable over time. Whole root system conductance did not significantly differ between genotypes, but a significant increase with time was observed (*P* < 0.001) (Table 3).

**Table 3.** Summary of measured total root length and simulated SUF per soil horizon as well as modeled whole root system conductance for the 3 grapevine rootstock genotypes (“101-14”, “SO4”, and “R110”) at 2 time points (T1: 3 months after planting, T2: 6 months after planting). Values are presented as mean ± SD. Statistical significance was assessed using a mixed effects model with genotype and time as fixed factors and block as a random factor. Significance levels are indicated as follows: n.s. (not significant), ^.^ (*P* < 0.1), * (*P* < 0.05), ** (*P* < 0.01), *** (*P* < 0.001). Letters (a, b, c) denote significant differences between genotypes based on post hoc pairwise comparisons. No significant effects associated with block were detected for any of the parameters.

		101-14			SO4			R110					
Parameter	Soil horizon (cm)	T1	T2		T1	T1	T1	T1	T2		Rootstock	Time	Interaction Rootstock:Time
Measured total root length per soil horizon (cm)	0–30	568 ± 84	436 ± 148	b	579 ± 223	724 ± 369	c	324 ± 144	283 ± 118	a	***	n.s.	n.s.
	31–60	508 ± 120	1,188 ± 242	ab	415 ± 188	982 ± 113	a	741 ± 308	1,018 ± 264	b	*	***	*
	61–90	2 ± 3	314 ± 151	a	0 ± 0	280 ± 184	a	2 ± 4	492 ± 149	b	*	***	*
	91–120	0 ± 0	22 ± 40	a	0 ± 0	17 ± 20	a	0 ± 0	162 ± 111	b	***	***	***
Modeled SUF per soil horizon	0–30	0.39 ± 0.14	0.16 ± 0.08	b	0.42 ± 0.16	0.24 ± 0.11	b	0.23 ± 0.10	0.07 ± 0.03	a	***	***	n.s.
	31–60	0.61 ± 0.13	0.64 ± 0.07	–	0.58 ± 0.16	0.59 ± 0.14	–	0.76 ± 0.09	0.53 ± 0.05	–	n.s.	·	**
	61–90	0.00 ± 0.00	0.18 ± 0.07	a	0.00 ± 0.00	0.16 ± 0.10	a	0.00 ± 0.00	0.29 ± 0.04	b	***	***	***
	91–120	0.00 ± 0.00	0.01 ± 0.02	a	0.00 ± 0.00	0.01 ± 0.01	a	0.00 ± 0.00	0.09 ± 0.05	b	***	***	***
Root system conductance (cm^3^ cm^−1^ d^−1^)	Total	0.03 ± 0.01	0.09 ± 0.02	–	0.02 ± 0.01	0.08 ± 0.02	–	0.03 ± 0.01	0.09 ± 0.02	–	n.s.	***	n.s.

### Simulation of grapevine RSA

The simulation of RSA using the CPlantBox model effectively captures the genotypic differences among the 3 grapevine rootstocks. Key model input parameters, derived from the digitized root systems of our T2 excavation (6 months after planting), are detailed in Table 4. For instance, maximal root length ($l_{\max}$) for type 2 roots varied among the genotypes, with “R110” exhibiting the longest roots (110.1 cm) compared to “101-14” (99.5 cm) and “SO4” (89.9 cm). Initial elongation rates (*r*) and insertion angles (θ) were also genotype specific, reflecting different growth dynamics and determinants of RSA establishment.

**Table 4.** Model input parameters (mean ± SD) for the CPlantBox simulation of RSA for the 3 grapevine rootstock genotypes (“101-14”, “SO4”, and “R110”). Parameter estimation was performed based on digitized 3D data from T2 (24 root systems, 6 months after planting). Tropism probability is based on the spatial distribution of type 2 root tips, indicating the likelihood of roots exhibiting plagiotropic (0) or gravitropic (1) growth behavior.

			101-14		SO4		R110	
Description	Parameter name	Units	Type 2	Type 3	Type 2	Type 3	Type 2	Type 3
Maximal root length	$l_{\max}$	cm	99.5	47.2	89.9	66.7	110.1	77.4
Initial elongation rate	*r*	cm	0.28	0.07	0.29	0.08	0.37	0.10
Length of basal zone	$l_{b}$	cm	8.4 ± 6.2	–	7.8 ± 5.9	–	11.9 ± 10.6	–
Length of apical zone	$l_{a}$	cm	11.8 ± 8.3	–	16.4 ± 9.4	–	25.6 ± 16.9	–
Interlateral distance	$l_{n}$	cm	4.1 ± 3.2	–	4.8 ± 3.5	–	6.7 ± 6.4	–
Insertion angle	θ	rad	54.4 ± 20.6	57.6 ± 21.9	51.8 ± 22.5	50.4 ± 22.5	54.9 ± 23.0	51.46 ± 22.5
Root radius	*a*	cm	0.23 ± 0.04	0.05 ± 0.02	0.20 ± 0.04	0.05 ± 0.02	0.22 ± 0.03	0.05 ± 0.02
Tropism type	Type	$\left\{0;1\right\}$^a^; probability	0 (0.15); 1 (0.85)	1	0 (0.35); 1 (0.65)	1	0 (0.05); 1 (0.95)	1
Tropism strength	*N*	1	2	1	2	1	2	1
SD of random angular change	σ	cm	0.2	0.2	0.2	0.2	0.2	0.2

^a^ 0 = plagiotropism; 1 = gravitropism.

**Fig. 8.**
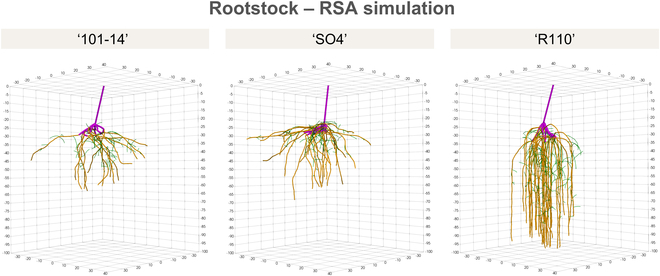
Simulation output of RSAs for the 3 grapevine rootstock genotypes (“101-14”, “SO4”, and “R110”) using the CPlantBox model. The simulations were conducted over a period of 180 days, starting from the initial static root system. The purple color represents the initial static root system, including the stem (type 0) and 1-year-old adventitious roots (type 1). First-order lateral roots (type 2), which emerge from static adventitious roots, are shown in brown. Second-order lateral roots (type 3), corresponding to roots branching from type 2 roots, are displayed in green. Simulated RSA patterns closely align with the empirical digitized root systems observed at 6 months after planting, demonstrating the model’s accuracy in capturing the dynamic development of root architectures.

The simulation outputs, presented in Fig. 8, demonstrate a high degree of similarity to the original digitized root systems, validating the model’s accuracy. Our probabilistic tropism function accurately represents the root growth patterns observed in our empirical data. For example, “R110” roots predominantly exhibited gravitropic growth (probability = 0.95), aligning with its pronounced vertical rooting behavior, while “101-14” and “SO4” showed higher probabilities for plagiotropic growth (probabilities = 0.15 and 0.35, respectively), capturing their more extensive lateral spread of type 2 roots. The additional model parameters for root tropism, including the strength of tropic responses (*N*) and the SD of random angular changes (σ), were fine-tuned to match the observed data, but were set to identical values across all 3 genotypes. Our approach demonstrates the robustness of CPlantBox in simulating genotype-specific RSA development, providing a powerful tool for predicting root growth of perennial species.

## Discussion

The development and refinement of RSA models over the past decades have been fundamental in advancing the understanding of root functioning, particularly in nutrient and water uptake processes [44]. The availability of high-precision data from field experiments conducted in realistic agricultural settings greatly benefits such modeling approaches. The assessment of root traits such as emergence, elongation, branching patterns, growth angles, or secondary growth modifications in a meaningful resolution under natural conditions, however, is challenging. Our proposed pipeline addresses this challenge by facilitating the acquisition of high-resolution RSA data and its transformation into the interoperable RSML format, thus enabling direct integration into existing root growth models and significantly advancing the capacity to simulate and understand RSA in real-world conditions. We chose different rootstock genotypes of the grapevine, representing woody perennial root systems with high complexity, to demonstrate the flexibility of the proposed approach. 3D RSA models have predominantly been parameterized for annual crops, with only a few exceptions (e.g., [45]).

In comparison to other root system analysis methods (as for instance discussed by [46]), the proposed method of field excavation combined with in situ 3D digitization presents a novel approach toward the architectural analysis of woody root systems. It allows for a spatially accurate representation of root architecture in its natural environment, overcoming limitations associated with traditional in situ and ex situ methods like soil coring, minirhizotrons, or pot experiments in general. Unlike pot or greenhouse experiments, which may not fully capture the complexity and natural growth patterns of root systems, field experiments provide an in-depth view of root architecture without the constraints of containerized environments. Compared to nondestructive methods such as rhizotrons or minirhizotrons, our methodology is much more labor-intense (necessitating approximately 2 person-days per root system) and potentially destructive. Furthermore, while 3D digitization offers precise spatial data on root architecture, it may not capture fine root details as effectively as some high-resolution imaging techniques used in controlled settings. Each method has unique advantages tailored to specific research objectives. Soil coring offers simplicity and cost-effectiveness for assessing root biomass and production in situ across fields, but fails to account for nonuniformities of root distributions in the horizontal plane, and its depth resolution is limited. Additionally, soil coring requires a large number of samples to accurately depict spatial rooting patterns and involves substantial time investment for post-sampling processing, including rinsing, sorting, and scanning the roots [47]. While soil trench profiles offer an accessible means for observing 2D root distribution and morphology directly in the field, they are constrained by their limitation to more easily identifiable, larger roots. For mapping coarse root architecture across large volumes, alternative nondestructive methods such as ground-penetrating radar (GPR) are emerging [48]. Recent advances also demonstrate GPR’s ability to detect fine roots in agricultural crops under specific conditions, such as Liu et al. [49], showing that GPR can predict root biomass and diameter in crops like wheat, making it a valuable tool for high-throughput root phenotyping. However, challenges remain, particularly with calibration across diverse soil types, moisture conditions, and detecting deep or densely distributed roots, as commonly found in grapevines or in mixed cropping systems such as cover-cropped vineyards. Our proposed method of field excavation followed by 3D digitization circumvents some of these limitations by capturing the spatial arrangement and detailed structure of root systems, preserving the natural root–soil interface. Despite its labor-intensive nature, our approach provides a valuable depth of fully annotated data, complemented by the precision of 3D digitization.

Digitization of excavated root systems can be achieved through various methodologies, including in situ 3D digitization, which we employed, as well as emerging approaches like LiDAR (Light Detection and Ranging) or laser scanning systems [50], or 2D scanning (e.g., RhizoVision; [51]) after root removal from the soil. While 2D scanning offers high-resolution imagery for finer root structures, these techniques face limitations, particularly in the absence of real-time annotation or potential occlusion of overlapping roots during scanning. This lack of annotation and the risk of root overlap often hamper the accurate reconstruction of the overall root topology and RSA, which is essential in complex field settings. By contrast, in situ 3D digitization allows for the preservation of the root’s natural spatial arrangement within the soil, making it highly advantageous for capturing the natural root architecture and correct topological structure of field-grown woody perennials. However, it is more labor intensive and may not match the resolution of 2D scanning systems for finer details. The choice of digitization method should be dictated by the specific research objectives. For high-resolution imaging of fine roots, 2D scanning may offer better detail, but for maintaining root architecture and studying large, complex root systems like those of grapevines, in situ 3D digitization remains indispensable. A potential solution to address these trade-offs is a combined approach, where in situ 3D digitization might be used to capture the overall coarse root architecture, followed by targeted 2D scanning of specific root segments. This combination could enhance the resolution of fine root branching details while simultaneously increasing phenotyping throughput.

The digitization method has been shown to be accurate and reliable for in situ digitization of root system within the soil matrix. While human error seemed a larger contributor to total digitization error compared to technical error, the low absolute errors relative to the size of the digitized root system show that the method is adequate for this application. As the root system’s topology is documented via the digitization protocol, the precise RSA reconstruction is possible with the described method. Our findings emphasize the importance of in situ digitization of RSA, offering a more natural portrayal of large root systems as opposed to digitization after extraction from the soil. Removal of the roots from the soil would lead to considerably biased root depth profiles and a misestimation of resource uptake in the investigated case of actively growing grapevine roots with limited diameter. Nevertheless, sporadic evidence collected during excavation in winter and on older root systems point to a higher stability of the roots after termination of the vegetative cycle and removal from soil after excavation has, for instance, been used for digitizing the structural roots of older woody perennials, such as 50-year-old *Pinus pinaster* [45]. The comparison between washout and excavation highlights the relevance of method selection based on the research objective. If accurate estimation of fine root length is essential, the washout method presents a clear advantage. Excavation, on the other hand, is faster and less resource intensive (no water wastage) while preserving the original root location in the soil. A realistic count of fine roots and interlateral branching distances of fine roots in situ can be obtained on excavated roots, confirming its utility in field-based root system research. The shortfall in measuring fine root lengths in excavation can be addressed in silico, where fine root lengths can be supplemented through computational models by integrating detailed and species-specific knowledge about fine root dynamics and lengths derived from other phenotyping methods, such as rhizotrons and washouts as well as from published data (e.g., [52]).

The current approach to analyzing grapevine RSA relies on manual excavation and in situ 3D digitization, which are labor intensive and require significant coordination from skilled personnel. These requirements pose challenges for scaling the methodology and applying it more broadly. To overcome these challenges, future research should focus on refining these processes to improve efficiency and feasibility. One promising solution are partial excavations in combination with virtual upscaling using models. Such partial extractions would support efficient upscaling of RSA studies in silico, particularly for genotypes with well-documented reference datasets. Furthermore, optimized sampling strategies—targeting key root system sections—combined with machine learning techniques could predict unmeasured root structures, thereby minimizing excavation efforts without compromising accuracy. Another promising strategy would be to explore semi-automation options to enhance the digitization process, particularly through voice-controlled annotation. Voice control would allow a single researcher to digitize and annotate simultaneously, eliminating the need for a second team member and expediting the process. This semi-automated workflow is a realistic step toward reducing resource demands while maintaining the high quality of collected data.

Despite the intensive workload required for root excavation and 3D digitization, the application of this method remains feasible for various woody perennial crops, particularly grafted crops such as fruit trees (e.g., *Prunus* species) and other orchard systems, or tree species (e.g., *Pinus pinaster* [22] or *Pinus ponderosa* [50]). While the electromagnetic field approach is limited by depth and horizontal spread of root systems in terms of spatial range and resolution of the transmitter (e.g., maximum range of 3 m for the Polhemus Fastrak system), this method offers a comprehensive solution for species with complex root architectures. In cases where deeper or more extensive root systems are present, adaptations such as partial extraction combined with computational upscaling techniques or aligning multiple 3D point clouds using landmarks could mitigate these limitations. Further limitations arise when conducting experiments that require large-scale infrastructure, such as extensive field phenotyping platforms or systems for environmental or crop manipulation, which often involve extensive support structures and power lines. These setups may pose challenges due to electromagnetic interference. However, despite these potential barriers, the Polhemus system has been successfully used under such conditions, including in a free-air carbon dioxide enrichment (FACE) system, as demonstrated by Schmidt et al. [53]. Moreover, certain environmental conditions may hinder digitization, such as high precipitation zones (e.g., tropical or monsoon regions), saturated soils, or densely packed root networks. Nevertheless, these limitations can often be addressed through *in silico* simulations. High-resolution data from this method also provide an invaluable resource for validating scalable techniques such as soil coring or GPR, enabling wider applicability across various environments [28]. Once models are parameterized with genotype-specific empirical data, virtual upscaling can simulate root system development under diverse conditions, allowing the method to be extended to a broader range of field experiments and cropping systems. This adaptability makes it particularly effective for use in crop breeding programs and genotype–environment interaction studies.

Rootstocks play a pivotal role in grapevine physiology and adaptation to environmental stresses [54–56]. For instance, the utilization of drought-tolerant rootstocks is considered as sustainable adaptation strategy to withstand declining soil water availability in viticultural areas, as predicted under the context of global climate change [57,58]. However, scientific understanding of genotype-specific root traits under field conditions is still scarce for most grapevine rootstocks [13]. In this regard, our proposed methodology demonstrated efficacy in detecting genotypic differences in grapevine RSA. We have shown that genotype effects on RSA development are detectable within the first year of grapevine establishment, but our findings also underscore the importance of root system age in the development of RSA characteristics, with notable genotypic traits becoming detectable primarily between 3 and 6 months after planting. For instance, the genotype “R110” consistently exhibited fewer lateral roots than “101-14” and “SO4”, which might indicate a genotype-specific growth strategy that prioritizes fewer, but possibly more efficient, lateral roots for resource acquisition. Its pronounced deep rooting behavior and water uptake efficiency suggests a potential advantage for drought resilience and resource acquisition in deeper soil profiles, which aligns well with published data on grapevine rootstock drought tolerance and rooting pattern (e.g., [55,59,60]). As root systems with similar overall root length density (RLD) may exhibit very different root distribution within soil compartments, our 3D approach offers significant advantages over 2D methods [61]. For instance, the root system width, height, and convex hull volume have been shown to be significantly different across the rootstock genotypes, which may affect water uptake capabilities in cropping systems.

Our simulation approach successfully adapted the CPlantBox model to accommodate grapevine root systems, reflecting practical viticulture scenarios where rooted cuttings are planted using mechanical planting machines. By integrating an initial static root system, we effectively represented the typical starting conditions in commercial vineyards. The simulations produced root systems that closely aligned with the original digitized root systems observed in our empirical data, demonstrating the accuracy and efficacy of our model. A notable feature of our simulation approach is the implementation of a probabilistic model for root tropism, where emerging type 2 roots have a specific likelihood of exhibiting plagiotropic growth behavior instead of gravitropic orientation. Specifically, we employed our probabilistic tropism function to mimic the anchorage roots primarily observed in the “101-14” and “SO4” rootstocks. This approach could accurately display genotypic differences in tropism responses for the same root type, solely based on topological information. This flexibility in CPlantBox highlights its ability to accurately simulate the root architecture of different genotypes, providing a powerful tool for predicting the root growth of woody perennials. While our simulations have shown promising results, there are several areas for future improvement. For instance, incorporating more observational data to model secondary growth modifications or root turnover rates will be crucial for developing a perennial model that accurately represents the dynamic nature of root systems over time. This is particularly important for perennial species like grapevines, where root systems continue to develop and change over multiple growing seasons. Adapting our model for perennial root system development will also likely involve the integration of higher-order branching and the potential transition of absorptive roots into structural roots. For instance, our results showed a significant decrease in the number of type 1 roots between 3 and 6 months after planting, which could be an indicator of turnover or the likelihood of specific root types to turn into perennial root structure.

Despite successful simulations, our proposed model currently neglects root–soil–feedback processes and a dynamic representation of grapevine RSA in response to heterogeneous soil properties requires further exploitation [62]. Dynamic feedback loops, where the heterogeneity of soil influences root growth and root development modifies soil properties through water and nutrient extraction, present fundamental challenges for existing modeling frameworks [63,64]. Ideally, models should be capable of dynamically representing RSA adaptations in reaction to the spatial and temporal variations of soil properties found in realistic agricultural scenarios. Specifically for grapevines, integrating models that incorporate viticultural soil water budgets—considering factors such as slope, planting density, and cover crop management (e.g., [65])—becomes imperative. Combining both, root and soil modeling, is essential for generating realistic viticultural scenarios, which can significantly enhance the predictability and utility of RSA adaptations in agronomic practices (e.g., in respect to cover crop management). Future directions should also involve the integration of aerial parts to achieve a comprehensive whole-plant model, such as combining grapevine root models with shoot models (e.g., “Virtual Riesling” [66]) or integrating species-specific shoot architectural data into whole-plant modeling frameworks such as CPlantBox. Employing platforms capable of modeling both roots and shoots would ensure seamless developmental coordination and avoid complexities of merging different models. Especially for modeling water-related processes (e.g., transpiration), interconnecting below- and aboveground plant processes would enhance the model applicability across agricultural practices. Moreover, advancing computational root models to simulate crop-scale environments (e.g., grapevine row crops) and intercropping is essential. These adaptations would allow models to reflect the realities of viticulture, where cover crops are increasingly utilized for sustainable farming. Exploring interspecific interactions through modeling could help in identifying more productive and environmentally friendly agricultural practices [19].

## Conclusion

This study presents a comprehensive methodology that combines field excavation, in situ 3D digitization, and modeling to analyze and simulate the RSA of woody perennials. By applying our methodology, we have successfully captured and quantified RSA dynamics across different grapevine rootstock genotypes. Key findings from our field excavations revealed substantial genotypic differences in RSA parameters and water acquisition capabilities. The high-resolution RSA data acquired through in situ digitization highlight the importance of preserving natural root structures for accurate modeling and interpretation. These data not only enhance our understanding of the spatial and temporal dynamics of root development but also enable the parameterization of root growth models. We have adapted the CPlantBox model to include an initial static root system, marking a significant advance in accommodating the unique conditions of rooted cuttings in viticulture. Future enhancements of our approach should focus on integrating longitudinal data from mature grapevines and vineyard water balance models to simulate diverse drought scenarios. Expanding the model to include whole-plant dynamics and environmental interactions will further increase its predictive accuracy and practical utility in viticulture.

Our pipeline advances root system research by combining detailed phenotyping and modeling, setting a robust foundation for developing RSA ideotypes that enhance water uptake efficiency and contribute to the sustainability of woody perennial crops under changing climatic conditions.

## Data Availability

Data collected and/or analyzed during this study can be obtained from the corresponding author upon reasonable request.

## Supplementary Materials

Supplementary 1 Sections S1 and S2 Figs. S1 to S7
