# Supplementary material for: A Field-to-Parameter Pipeline for Analyzing and Simulating Root System Architecture of Woody Perennials: Application to Grapevine Rootstocks
Source: Plant Phenomics. 2024 Dec 11;6:0280. doi: 10.34133/plantphenomics.0280 (PMC11633832; doi:10.34133/plantphenomics.0280)
Supplement: Supplementary 1 — Sections S1 and S2 Figs. S1 to S7 [file plantphenomics.0280.f1.docx]

# Supplementary Materials for:

# A Field-to-Parameter Pipeline for Analyzing and Simulating Root System Architecture of Woody Perennials: Application to Grapevine Rootstocks

# Lukas Fichtl^1*^, Daniel Leitner^2^, Andrea Schnepf^2^, Dominik Schmidt^3^, Katrin Kahlen^3^, Matthias Friedel^1^

^1^Department of General and Organic Viticulture, Hochschule Geisenheim University, Geisenheim, Germany.

^2^Forschungszentrum Juelich GmbH, Agrosphere (IBG-3), Juelich, Germany.

^3^Department of Modeling and Systems Analysis, Hochschule Geisenheim University, Geisenheim, Germany.

*Address correspondence to: Lukas.Fichtl@hs-gm.de

**S1: Guideline for RSA Excavation, Digitization and Data Post-Processing Pipeline (page 2–7)**

**S2: Figure Tropism Quantification (page 8)**

**S1: Guideline for RSA Excavation, Digitization and Data Post-Processing Pipeline**

The following workflow outlines the methods employed for *in situ* root system excavation, 3D-digitization, and subsequent data processing for root system analysis of grapevine rootstocks. This pipeline is flexible and may be adapted for similar studies involving woody perennials.

1. **Field Trial Setup and Planting**
   The design of the field trial should align with the specific research objectives, whether focused on plant competition, root architecture assessments, or responses to environmental factors. It is advantageous to plan excavation campaigns during the trial setup to ensure appropriate spacing, replication, and access for root excavation. For crops such as grapevines that rely on trellis systems, the use of non-metallic materials (e.g., wooden posts, bamboo stakes, plastic wires) is advised to mitigate electromagnetic interference that could affect the precision of the 3D root digitization process, when using a digitizer that operates on electromagnetic principles.

**
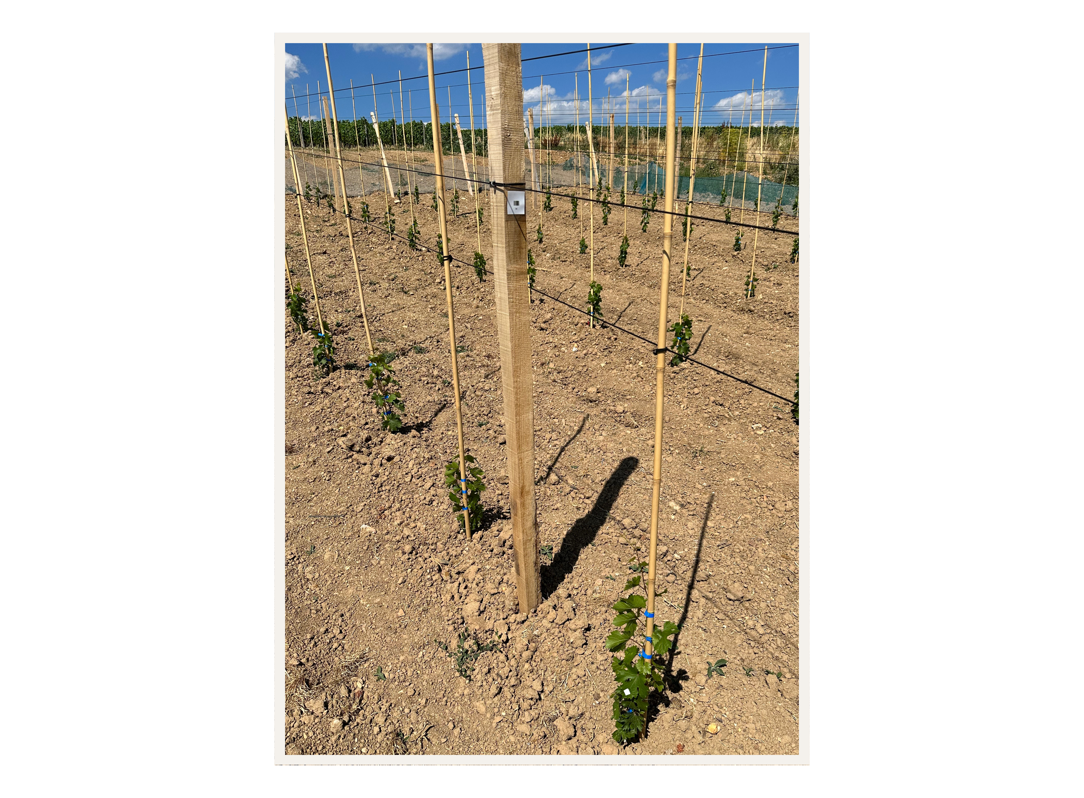
**

Figure 1: Trellis system used in the field experiments, developed to be completely metal-free in order to avoid electromagnetic interference during the digitization process. The setup includes wooden posts, bamboo planting sticks, and plastic wires, providing a stable and interference-free environment for root system excavation and digitization.

1. **Root System Excavation**
2. *Stabilization and Excavation Preparation*

Prior to excavation, stabilizing the plant stem is necessary to maintain its original elevation relative to the soil surface. This may be achieved by securing the stem to a horizontal framework (e.g., attaching it to a wooden stick). In cases where the shoot architecture is of interest, it is recommended to first digitize the above-ground parts before cutting the plant near the grafting point to facilitate excavation.


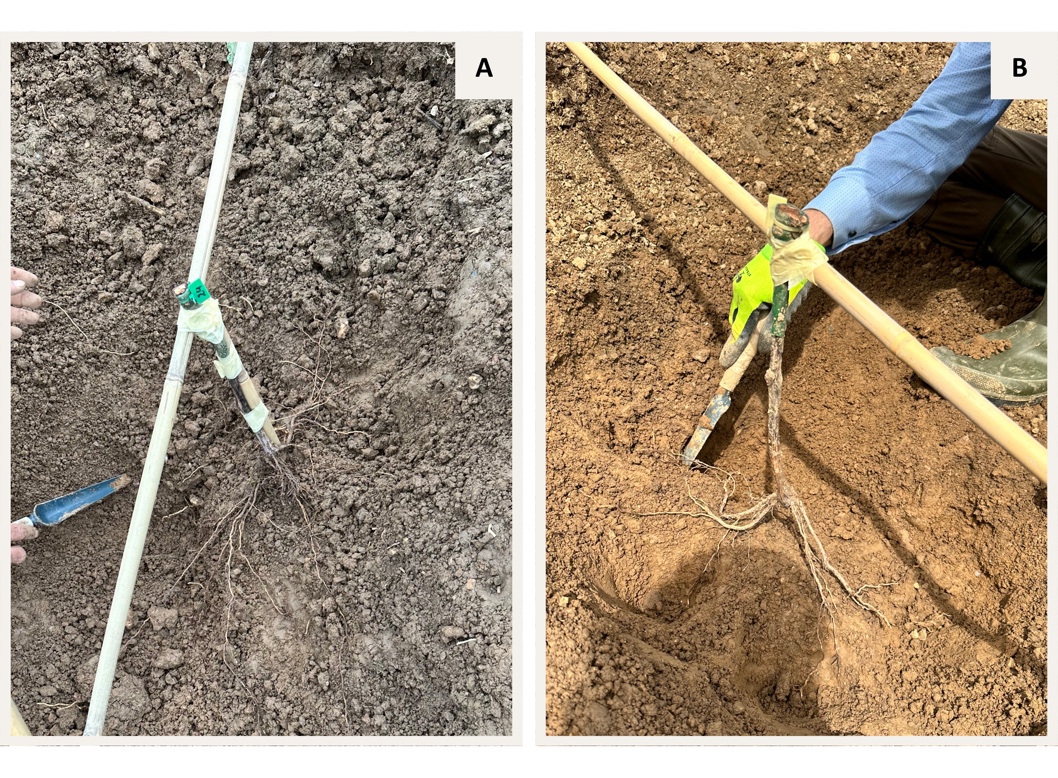


Figure 2: (A) and (B) illustrate the initial stages of the root system excavation. In both images, the grapevine shoot system has been cut at the grafting point, and the stem is stabilized using a horizontal wooden stick, which is secured on both sides of the vine by placing it into the soil. This stabilization helps to maintain the original position of the vine during excavation. The process begins by carefully exposing the stem down to the point of adventitious root initiation to estimate the general direction of root distribution. The images also show the use of weeding trowels, the preferred tool for gently loosening soil near the roots during the excavation.

For ease of access, a trench may be excavated near the plant but positioned far enough away to avoid damaging the roots, ensuring an ergonomic work position during the excavation process. It is advisable to dig preliminary trenches around a few plants to estimate root system spread and determine appropriate trench placement. In our study, the trench was dug approximately 1 meter away from the plant between rows, and it was observed that the top 20 cm of soil were typically root-free due to the planting of cuttings, enabling additional careful removal of topsoil before detailed excavation.


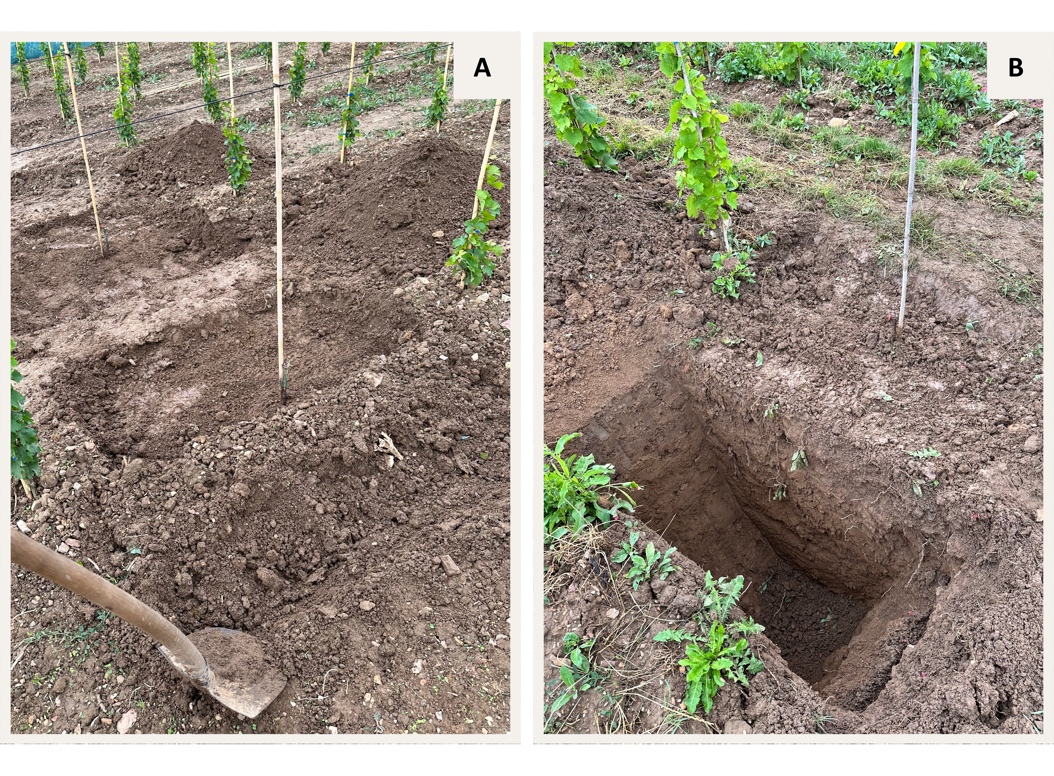


Figure 3: (A) shows the removal of the topsoil (approximately 20 cm) by hand using a shovel, around a 1 square meter area around the vine. This step ensures that the surface soil is carefully removed without damaging the root system. (B) shows the trench, which was dug approximately 1 meter away from the vine in the middle of the row using an excavator. The trench was excavated to a depth of around 1.5 meters to allow a healthy work position for further excavation of the root system. In this case, we used an excavator with a 40 cm wide bucket to dig the trench.

1. *Excavation of Root Systems*

Excavation begins by exposing the vine stem down to the adventitious root initiation zone to assess the overall root system orientation. Individual lateral roots and their branches are then carefully excavated. The choice of excavation tools depends on soil conditions (e.g., moisture, texture), with garden forks, shovels, and spades being suitable for loosening non-rooted soil. For precision work near roots, specialized tools such as the “weeding trowel” are recommended for gently loosening the soil, while brushes can be employed under dry conditions to further expose roots without causing damage.


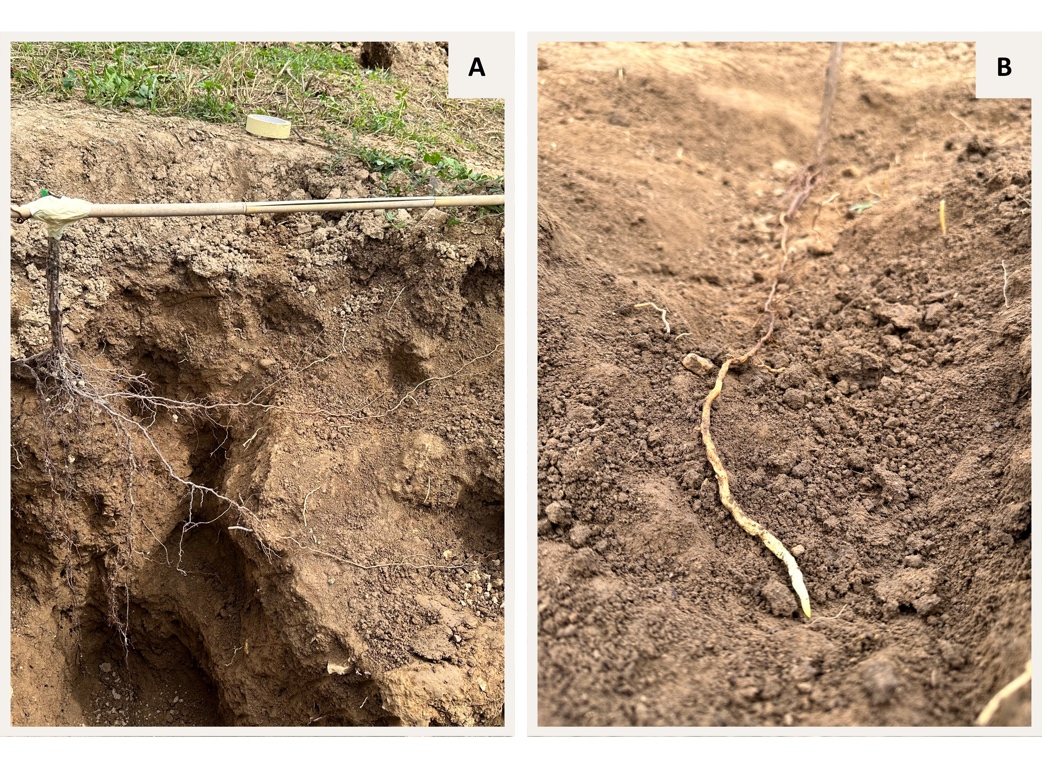


Figure 4: A) Example of a root system with extensive horizontal root spread. This shows how horizontal roots can remain supported in their original position after careful soil removal. B) Close-up of a horizontally growing root, demonstrating that the root maintains its natural position due to the careful support of the surrounding soil. For such horizontally oriented roots, we recommend fully exposing and digitizing them before excavating deeper or underlying roots to ensure accurate documentation and avoid confusion during 3D digitization.

Where possible, horizontal roots should be fully digitized before deeper excavation begins to avoid confusion in densely rooted areas and to prevent double-digitization. Measuring the diameter of individual roots with calipers at this stage ensures precise recording of root dimensions, which are then linked to the corresponding digitized root IDs.


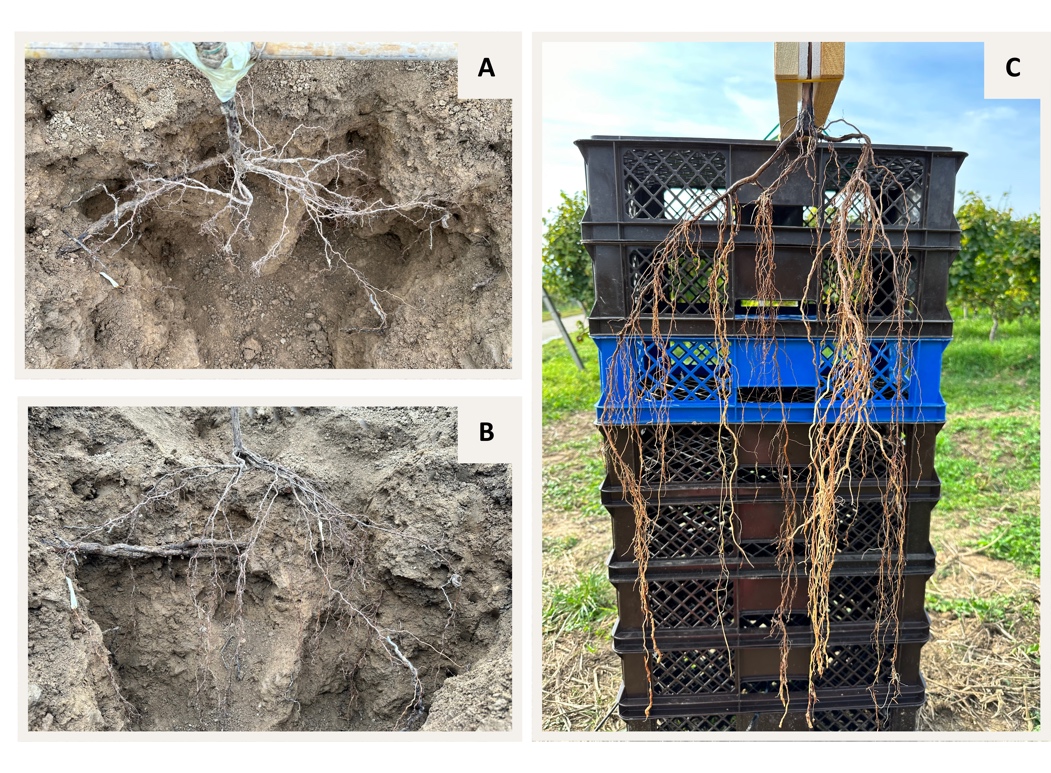


Figure 5: A) and B) Examples of fully excavated root systems maintaining their natural orientation in the soil. The roots are still supported by the surrounding soil structure, ensuring accurate digitization. C) Example of a root system in a hanging state after removal from the soil, demonstrating the significant loss of structure. Roots are not rigid enough to retain their original orientation, which emphasizes the importance of digitizing them while still in the soil to preserve their natural architecture.

1. *Handling of Damaged Roots*

In cases where roots are accidentally severed during excavation, they may be reconnected using tape, allowing the digitization process to proceed without significant disruption.

1. **3D Root System Digitization**
2. *Equipment Calibration*

A 3D electromagnetic digitizer (e.g., Polhemus Fastrak) is used for recording root coordinates *in situ*. Prior to each session, the system should be calibrated by measuring known distances and comparing the Euclidean distances of the 3D digitized data with the actual values. This process should be repeated under field conditions to account for potential electromagnetic interference.

1. *Digitization Protocol*

The digitization procedure begins at the base of the stem and follows the branching pattern of the adventitious, lateral, and fine roots. The transmitter should be positioned approximately 60 cm above the soil surface and secured using a stable, non-metallic frame. It is important to record branching points accurately to maintain continuity between lateral roots and their parent structures. Each root should be digitized using enough points to capture changes in growth direction and branching angles.


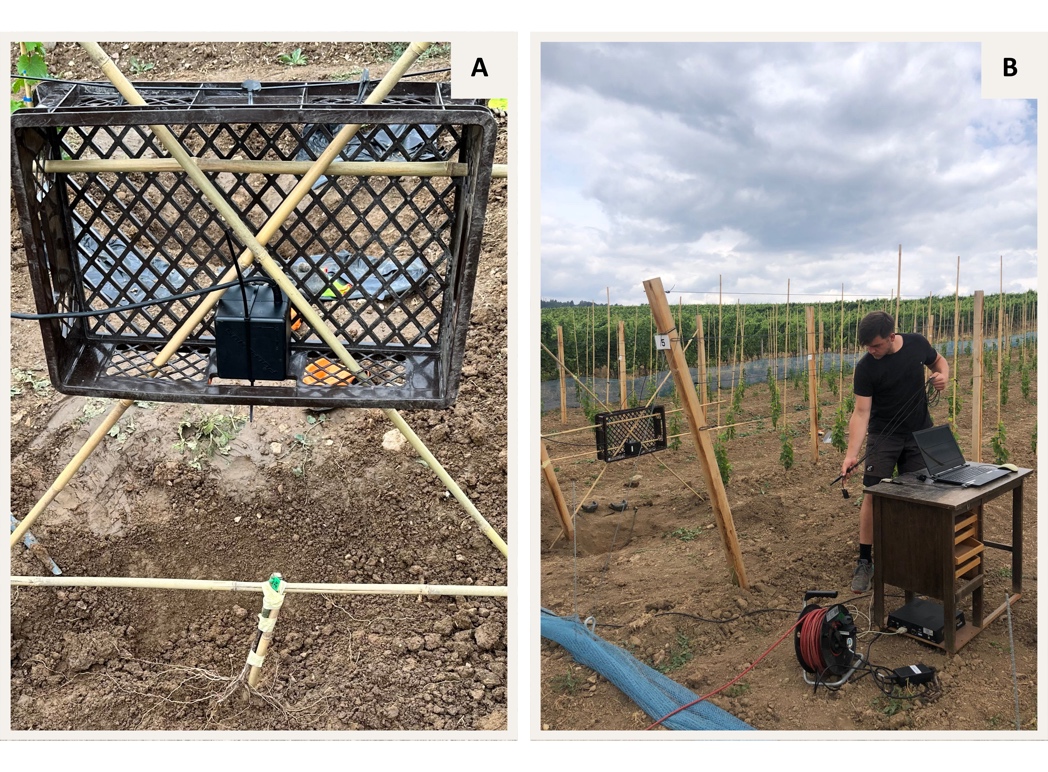


Figure 6: A) Custom-built support structure to stabilize the transmitter directly above the root system during digitization. The wooden sticks are reinforced with cable ties attached to the trellis system, providing stability for the transmitter. B) Full digitization setup, where the transmitter is connected to a laptop and main unit. The main unit is also connected to the pointer used for root system digitization.

1. *Real-Time Annotation*

Real-time functional and topological annotation is recommended using dedicated software (e.g., DigiTool). Clear protocols for annotating roots during digitization should be developed to allow for unambiguous reconstruction of the root system. Test runs are recommended to familiarize the team with the process and ensure consistent, accurate data collection.

1. *Potential Challenges*

Unfavorable weather conditions, such as rain, can disrupt the digitization process. In such cases, a metal-free pavilion may be used to protect the equipment, although this may be impractical in plots with high trellis systems. A portable power supply, such as a battery, can be utilized to maintain equipment functionality in remote field conditions.

1. **Data Processing**
   Data from the digitization process is exported as simple text files containing the x, y, and z coordinates, along with associated organ types and unique IDs that define the root system’s topology. The exported data can be processed and analyzed using various platforms (e.g. R). It is advisable to convert the data into an interoperable format, such as RSML (Root System Markup Language), which is supported by a range of phenotyping and modeling software platforms. In our study, the CPlantBox framework was used for model simulations and analysis of root system architectures. RSML files contain detailed geometric and root segment properties, enabling comprehensive evaluation of root architecture. Basic analyses can be performed using the CPlantBox *viewer* and *estimator*, with slight adaptations made for woody perennial characteristics.

**S2: Tropism Quantification**


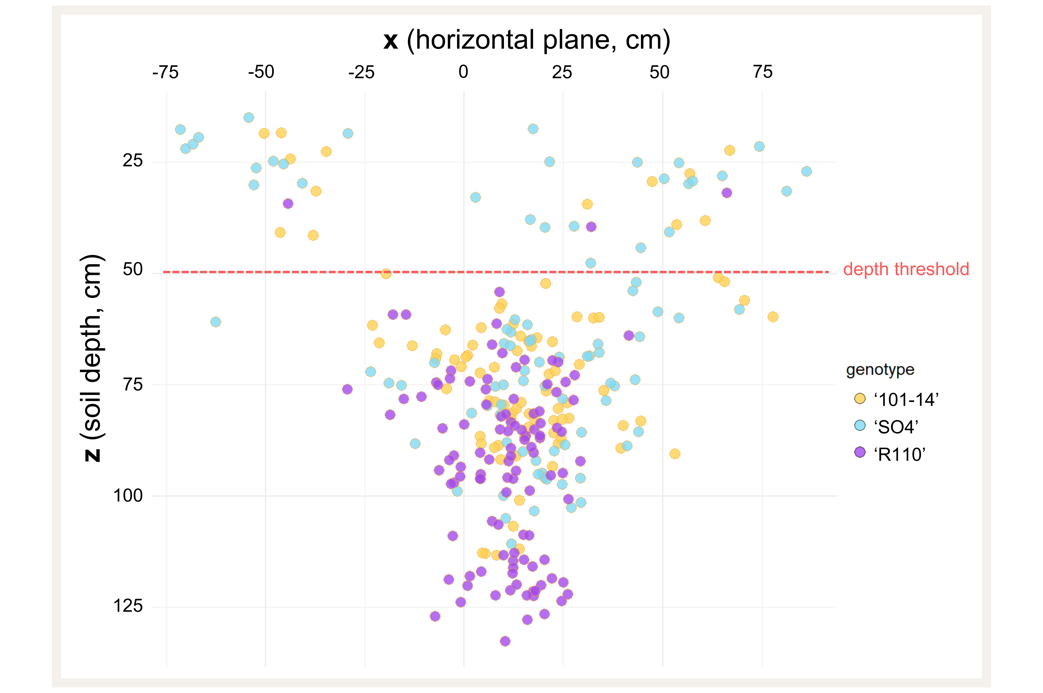


Figure 7: Spatial distribution of type 2 root tips for three grapevine rootstock genotypes ('101-14', 'SO4', and 'R110') six months post-plantation. Each point represents the position of a root tip from "long" type 2 roots (root length > 40 cm) in relation to soil depth (z-axis) and horizontal plane (x-axis). A soil depth threshold of 50 cm (indicated by the red dashed line) was used to differentiate between plagiotropic and gravitropic growth tendencies. Root tips located above 50 cm are classified as plagiotropic (horizontal growth), while those below 50 cm are classified as gravitropic (downward growth). Probabilities for each genotype's growth behavior were calculated as the percentage of root tips found in the upper (plagiotropic) or lower (gravitropic) soil layers.
